# The HIV-1 nuclear export complex reveals the role of RNA in CRM1 cargo recognition

**DOI:** 10.1016/j.molcel.2025.07.015

**Published:** 2025-08-21

**Authors:** Amber M. Smith, Yang Li, Arianna Velarde, Yifan Cheng, Alan D. Frankel

**Affiliations:** 1 Department of Biochemistry and Biophysics, University of California, San Francisco, San Francisco, CA 94158, USA; 2 Department of Biology, San Francisco State University, San Francisco, San Francisco, CA 94132, USA; 3 Howard Hughes Medical Institute, University of California, San Francisco, San Francisco, CA 94143, USA; 4 Lead contact

## Abstract

HIV utilizes host proteins to manipulate cellular processes during replication. One key interaction involves the HIV regulatory protein Rev with the highly conserved nuclear exportin chromosome region maintenance 1 (CRM1), which exports >1,000 proteins and ribonucleoprotein (RNP) complexes from the nucleus to the cytoplasm. Rev, with its associated viral RNA, is the first identified RNP cargo of CRM1. Here, we present the cryogenic-electron microscopy (cryo-EM) structure of the HIV-1 Rev/Rev response element (RRE)/CRM1/Ran nuclear export complex. A Rev dimer engages a unique CRM1 dimer at an uncharacterized cargo-binding site at the core of the complex, positioning the RRE within a charged pocket inside one CRM1 subunit. Direct contacts between the RNA and CRM1/Ran-guanosine triphosphate (GTP) highlight the critical role of the RRE. The structure, together with guided mutations and cellular assays, provides not only an unprecedented view of HIV RNA transport but also illuminates how CRM1 can recognize a diverse range of protein and RNP cargos.

## In brief

The HIV nuclear export complex shows that CRM1 recognizes diverse cargos through distinct sites, utilizing multipartite recognition via the NES and additional protein and RNA contacts. A unique CRM1 dimer, driven by Rev dimerization, facilitates direct interaction between CRM1/Ran-GTP and the RRE.

## Graphical Abstract

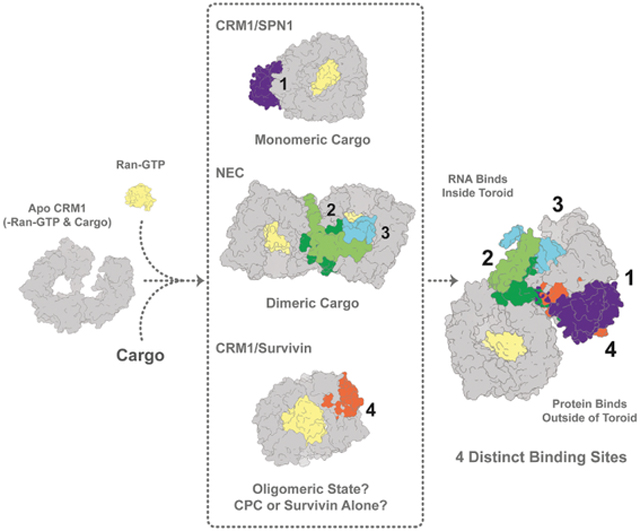

## INTRODUCTION

Like all viruses, HIV requires host protein interactions to redirect critical host biological processes during replication. HIV relies on overlapping reading frames and alternative splice sites to translate all the necessary proteins for viral replication and infection. A single ~9 kb viral transcript produced from the integrated viral DNA is fully processed (capped, spliced, cleaved, and polyadenylated) by the host cell machinery early in the viral life cycle to yield ~2 kb mRNA species encoding the Rev or Tat regulatory proteins or the Nef accessory protein.^1,2^ Later in the life cycle, HIV bypasses the host mRNA processing machinery to facilitate the nuclear export of intron-containing transcripts, which encode the remaining viral proteins and genomic RNA for packaging.^3,4^ In host cells, splicing and export of cellular mRNAs are tightly regulated in the nucleus, ensuring that intron-containing mRNAs are not exported to the cytoplasm.^5–7^ For such fully spliced mRNAs, export proteins are recruited to the mature mRNAs via the spliceosome and are then shuttled to the cytoplasm by the transporter associated with antigen processing protein (TAP).^8^ In HIV, intron-containing mRNAs are exported via another human export receptor, chromosome region maintenance 1 (CRM1).^9,10^ To export its incompletely spliced and unspliced transcripts, the HIV Rev protein^11–13^ functions as an adapter by binding simultaneously to the Rev response element (RRE),^14–16^ a structured region of the viral RNA located in a viral intron, and CRM1, forming an export complex.

Rev is comprised of 116 amino acids and has three functional domains: a bipartite oligomerization domain (OD; residues 12–26 and 50–60), an arginine-rich motif (ARM; residues 35–49) that functions as the RNA-binding domain and nuclear localization sequence (NLS), and a C-terminal nuclear export sequence (NES; residues 75–83) (Figure S1A). The Rev monomer adopts a hairpin structure, where the two ODs interact to form a hydrophobic core with an intervening helical ARM (Figure S1A).^17,18^ Rev dimerizes when two monomers interact via α or β faces in a V-shaped topology (Figure S1B) and can assemble into higher-order oligomers *in vitro* (Figure S1C).^17–21^ Approximately six Rev subunits bind to a truncated but functional 234-nt RRE, initiating at the high-affinity stem IIB site (Figure S2) to form discrete Rev/RRE ribonucleoprotein (RNP) complexes.^15,22–28^ In addition to the stem IIB site, which binds a Rev dimer,^20,22^ two other lower-affinity-binding sites—in stem IA and stem I—have been partially characterized.^15,26,29^ Maximal Rev-mediated nuclear export activity *in vivo* is observed with the full-length RRE (~355 nt), which includes the stem I site absent in the truncated RRE, whereas the shorter RNA retains >80% of Rev export activity and forms a biochemically stable complex.^15^ Current models suggest that Rev forms a hexamer on the truncated RNA, with the Rev molecules associating via protein-protein interactions,^26^ but the available dimeric Rev/IIB co-crystal structure,^20^ a small-angle X-ray scattering (SAXS) model of the RRE in the absence of Rev,^30^ and other biochemical studies are insufficient to describe the complete Rev/RRE assembly. Furthermore, it is unclear if the full-length RRE binds a precise number of Rev subunits *in vivo* or whether a distribution of stoichiometries exists.^15,26,31,32^ In this paper we examine the structure of the Rev/RRE complex in the context of the CRM1 export receptor.

Rev was one of the first proteins identified to contain an NES, a generally important feature for CRM1 interaction.^33–36^ CRM1, also known as Exportin-1 or XPO1, is a member of the karyopherin-β family and is responsible for the nuclear export of over 1,000 different human proteins and RNP complexes covering a diverse range of biological processes.^35–41^ CRM1 is the only known karyopherin (Kap) to export RNP cargos in addition to proteins, and, like other Kaps, it utilizes Ran as a cofactor for cargo-binding and release.^42^ CRM1 has a toroid-shaped structure formed by a ring of HEAT (huntington, **e**longation factor 3, protein phosphatase 2A, and **T**OR1) repeats, a common structural feature of all Kaps.^35,36^ However, unlike other Kaps, CRM1 binds its cargo on the outer surface of the HEAT repeats without directly interacting with Ran-GTP, as observed in several CRM1 structures solved with Snurportin-1 (SPN1).^43–45^ A major recognition feature of CRM1 cargos generally, including SPN1, is the docking of an NES peptide into a cleft formed by HEAT repeats 11 and 12, with considerable variation observed in the interactions with isolated NES peptides, depending on peptide length, conformation, and affinity.^46–50^ Besides the NES cleft and SPN1-binding site, additional protein-binding sites have been identified on the outer surface of the CRM1 toroid, including nucleoporin-binding sites (CRM1/Nup214, CRM1/Yrb2p, and CRM1/RanBP2)^45,51,52^ and Ran GTPase-activating protein (GAP)-binding sites.^51,53^ The interaction of CRM1 with SPN1 provides an interesting contrast with Rev, where a monomer of CRM1 binds to the monomeric SPN1^44^ while a dimer of CRM1 binds to the Rev/RRE RNP.^54^ This difference may be driven by the dimeric nature of Rev, the presence of RNA, or other factors. Understanding how CRM1 recognizes this important HIV RNP will not only provide insight into HIV replication but will also deepen our knowledge of CRM1 biology, addressing a long-standing question of whether RNA directly contacts CRM1.^35,42^

In this study, we report the structure of the HIV-1 nuclear export complex (NEC) using single-particle cryogenic-electron microscopy (cryo-EM). The structure reveals the importance of dimer recognition, previously unseen interfaces of human CRM1 with cargo, and how RNA engages the complex. The results demonstrate that, in addition to NES binding, multipartite interactions, including interactions with RNA, are critical to accommodate the expansive repertoire of cargos, both cellular and viral complexes. Guided by the NEC structure, we identified an alternative CRM1-binding site for an important host cargo, Survivin, suggesting the possibility of selective targeting of CRM1 interfaces for therapeutic intervention.

## RESULTS

### HIV-1 NEC structure description

**Figure 1.**
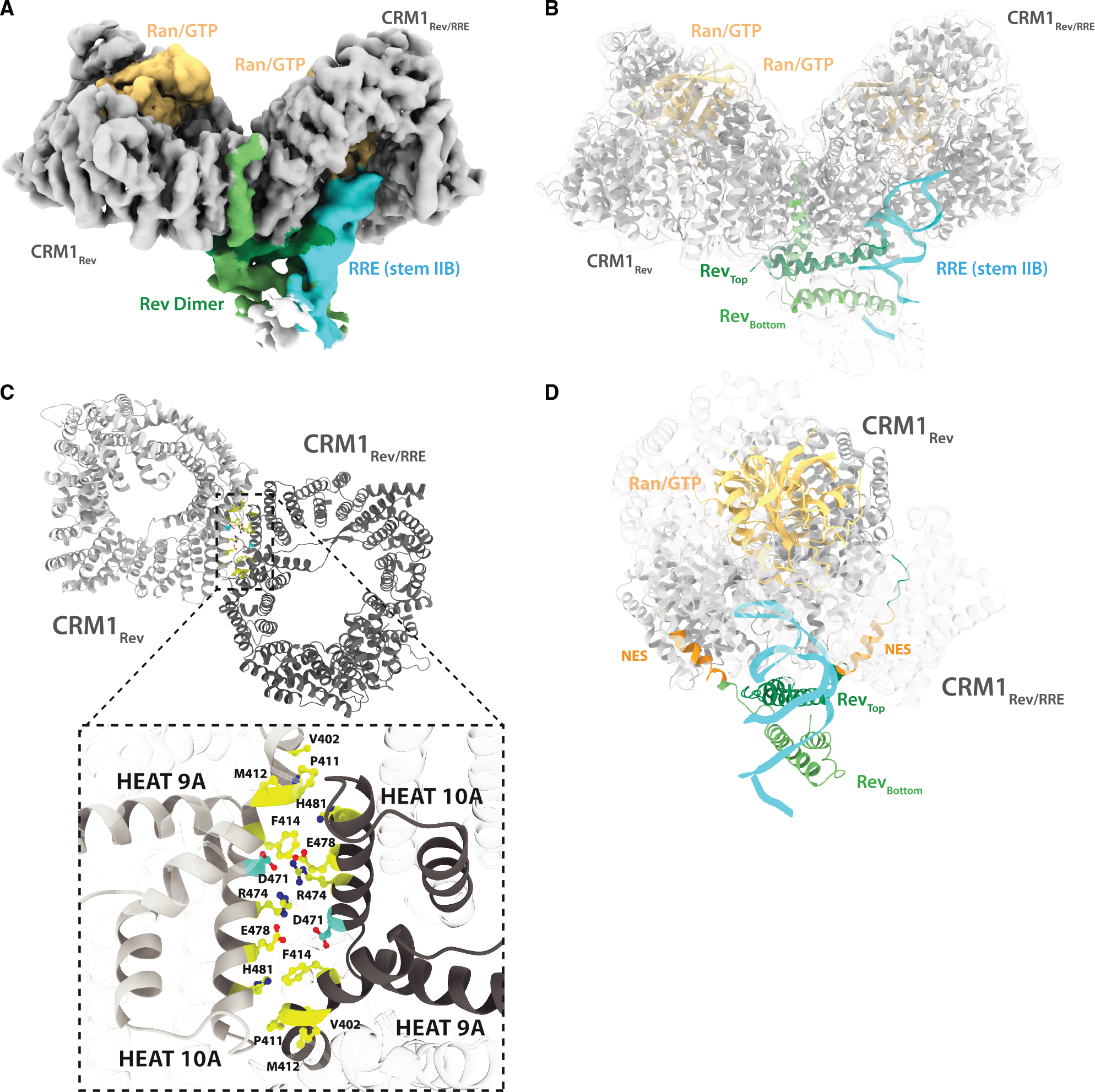
The HIV nuclear export complex (A) Cryo-EM reconstruction of the HIV-1 nuclear export complex (NEC), with individual components colored: CRM1 in gray, Ran-GTP in peach, RRE (stem IIB) in cyan, and two Rev subunits in light and dark green. (B) Ribbon diagram of the NEC with the same coloring shown in (A). (C) Ribbon diagram of the CRM1 dimer (two subunits in dark and light gray), highlighting higher primate species-specific residues critical for HIV-1 replication in yellow and a conserved aspartate residue in cyan. A close-up view of the CRM1 dimer interface reveals a polar network formed by the species-specific residues located in HEAT repeats 9 and 10 (V402, P411, M412, F414, R474, E478, and H481; shown in yellow) in combination with the conserved aspartate residue (D471; shown in cyan). (D) Side view of the NEC rotated 90° from the orientation shown in (A) and (B), with the CRM1 subunit closest to the viewer rendered transparent. This view highlights how each NES of the Rev dimer engages a separate CRM1 subunit on opposite sides of the CRM1 dimer.

Our prior work demonstrated that CRM1 dimerizes with the HIV Rev/RRE RNP,^54^ enabling us to use dimer formation to optimize NEC formation. While the complex appeared intact by negative stain EM, it consistently dissociated under cryo-EM conditions, limiting our ability to obtain a reconstruction of the intact complex. To generate an intact NEC suitable for cryo-EM studies, we modified Rev (HIV-1 HXB3 isolate), the RRE RNA (SF-2 isolate),^55^ and human Ran, ultimately using a Rev chimera with an engineered high-affinity NES (NS2Val) that facilitates CRM1 association even in the absence of Ran-GTP,^47^ an RRE variant (RRE_61_)^55^ that favors one of two observed RNA conformations predicted to adopt a five-stem loop structure over the four-stem conformation (Figure S2), which improved negative stain 2D class averages, and a Ran variant that combines two well-characterized modifications to prevent GTP hydrolysis.^56,57^ We also included a 113-amino acid phenylalanine-glycine (FG)-repeat polypeptide from the nuclear pore protein, Nup214 (residues 1,916–2,033), fused to maltose-binding protein (MBP), which has been shown to enhance CRM1 affinity for Rev.^45^ Altogether, the modifications overcame the dimer dissociation observed in cryo-EM and provided a suitable complex for NEC structure determination. Each modification maintained a structure indistinguishable from that formed with wild-type components as judged by negative stain (Figure S3), allowing us to obtain the HIV-1 NEC structure at a global nominal resolution of 4.2 Å, with a local resolution of 3.7 Å for the CRM1/Ran-GTP dimer (Figures 1A, 1B, and S4A; Table 1). The nominal resolution of the Rev/RRE portion is 5.8–6.8 Å, consistent with increased conformational flexibility as a function of distance from the CRM1/Ran dimer, as gauged by B-factors (Figure S4B). An atomic model of the CRM1/Ran-GTP/Rev-RRE complex (Figure 1B) was reliably produced by rigid-body docking of the crystal structures of CRM1/Ran-GTP^45^ and Rev/RRE^20^ followed by real-space refinement.^58,59^

The resolution of the CRM1/Ran-GTP dimer is sufficient to accurately trace the backbone with clearly resolved helical pitch, β-sheets, and some side-chain densities. The two CRM1 molecules are exactly the same with the exception of how one subunit engages Rev and the RRE. The residues that stabilize the CRM1 dimer are critical for Rev function and differ between primates and murines.^60,61^ We modeled these residues at the CRM1 dimer interface in a previous 25 Å structure,^54^ and the 3.7 Å resolution of our current NEC structure confirms that these residues located on HEAT 9A (V402, P411, M412, and F414) and HEAT 10A (R474, E478, and H481) create a highly polar interaction network flanked by hydrophobic residues that strengthen the human CRM1 dimer interface compared with its murine counterpart (Figures 1C and S5A).^54^

The NEC structure displays four major features: first, a single Rev dimer bridges the CRM1 dimer in a stacked arrangement, with the top Rev subunit making extensive contacts that further buttress the CRM1 dimer (Figure 1B). Second, the interaction between Rev and CRM1 occurs at a previously unobserved protein-protein interaction site on the outer surface of the CRM1 toroid. Third, the two NES peptides from the Rev dimer are able to span the CRM1 dimer using different conformations of a linker to dock within the two distant NES-binding pockets (Figure 1D). Fourth, Rev orients the stem IIB RNA to dock deeply inside one CRM1 subunit and make direct contacts to CRM1 and Ran-GTP (Figures 1B and 1D). Despite the dimeric nature of the interaction, the structures of the Rev dimer and interactions with CRM1 are highly asymmetric and explain many functional and biochemical aspects of the complex.

### Rev dimerization and β-face interactions

**Figure 2.**
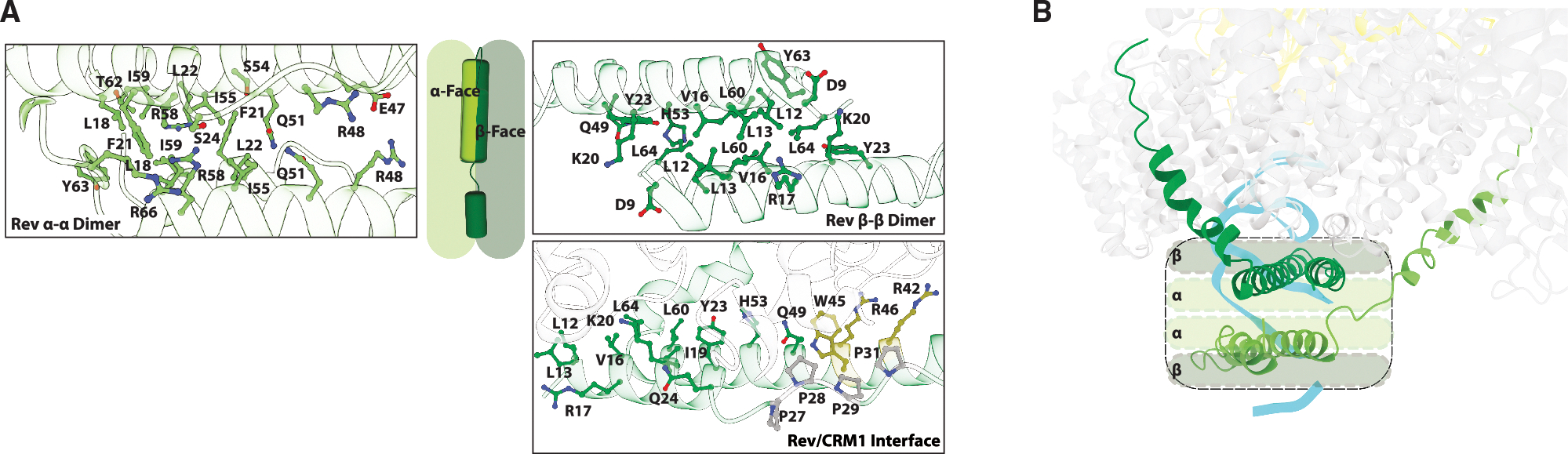
Rev engages proteins on both faces of its hairpin (A) Previous structural studies of Rev dimers have shown dimerization interactions occurring on both sides of Rev’s hairpin structure, utilizing solely its bipartite oligomerization domain (OD). Within the NEC, the entire β-face of Rev engages CRM1, with interactions extending beyond the OD to include residues within the hairpin turn (gray) and the arginine-rich motif (ARM: yellow), thereby orienting the Rev/RRE RNP. CRM1 is shown in white. (B) NEC structure showing that the Rev dimer stacks in a mirrored conformation beneath the CRM1 dimer, with their respective Rev dimer interfaces arranged perpendicularly. In this arrangement, the β-face of Rev either interacts with CRM1 or remains unoccupied, while the α-face of Rev forms the Rev dimer interface.

Numerous structural studies of Rev revealed that Rev can self-associate via both sides of its hairpin structure, utilizing hydrophobic residues within the OD (Figures 2A and S1C). The Rev dimer interface seen in the NEC is the same as in the crystal structure of a Rev-stem IIB RNA complex^20^ (Figure S6A) and in two apo-Rev structures.^17,19^ We refer to this side of the Rev hairpin as the α-face (Figures 2A and 2B). We previously showed that binding to stem IIB RNA repacks the hydrophobic residues of the α-face and changes the Rev dimer crossing angle to accommodate the RNA.^17,19,20^ Within the NEC, the core Rev dimer adopts the identical configuration and subunit crossing angle observed in a co-crystal structure with stem IIB (Figure S6A),^20^ indicating that this high-affinity site alone drives Rev into a defined configuration rather than utilizing the larger RRE framework to dictate its conformation. The opposite β-face mediates Rev-Rev interactions when the α-face is blocked by an antibody fragment^18^ or in the context of higher-order filamentous structures^21^ or during crystal packing.^17,19^ In the NEC, the β-face either interacts directly with CRM1 (Figures 1B and 2B) or is unoccupied. Rev-Rev interactions via the β-face were previously thought to be important for generating functional higher-order Rev oligomers on the RRE, but it is clear from the NEC structure that the β-face is critical for the interaction with CRM1, and we observe no evidence for any higher-order Rev-Rev interactions despite biochemical detection of more than two Rev molecules in the complex used for structure determination (Figure S6B). The interactions between the β-face and CRM1 span across both subunits of the CRM1 dimer and extend beyond the OD to include residues from the Rev hairpin turn and ARM (Figure 2A).

To further assess whether residues on the β-face may play a functional role in Rev-Rev association, we examined the behavior of a β-face mutant known to prevent binding of more than one Rev dimer to the RRE.^62^ The commonly studied L60R mutant blocks replication of the NL4–3 virus^63^ but still shows 30% of nuclear export activity in reporter assays.^22,63^ Negative stain 2D class averages of NECs assembled with Rev_L60R_ exhibited a mixture of monomeric and dimeric forms of CRM1, unlike the fully dimeric complexes observed with wild-type Rev (Figure S7). Thus, a single Rev dimer mediated by the α-face and bound to stem IIB is largely sufficient to engage the CRM1/Ran dimer, although maximal nuclear export requires more than one Rev dimer.^15^

### Rev-binding site on CRM1

**Figure 3.**
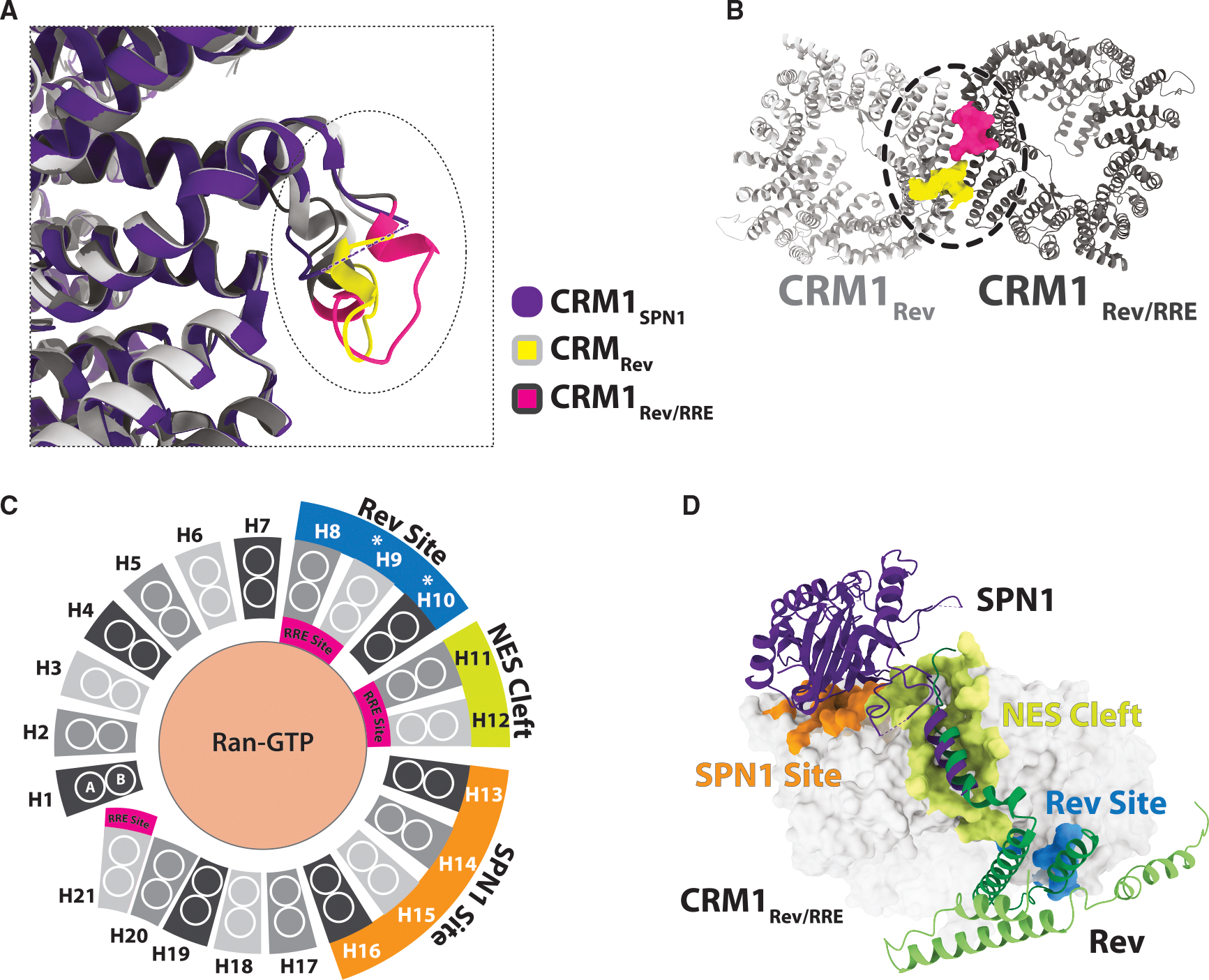
Rev binds to CRM1 at an uncharacterized protein site (A) In previous structures of human CRM1 (CRM1 shown in purple is from the CRM1/SPN1 complex; PDB:5DIS), a loop (residues 389–400) between HEAT repeats 8 and 9 is disordered. However, in the NEC, this loop is ordered in both CRM1 subunits (CRM1_Rev_ shown in light gray with a yellow loop, and CRM1_Rev/RRE_ shown in dark gray and a pink loop) and contributes to the Rev-binding site. (B) The ordered loops of CRM1 (ribbon diagram, CRM1 in light and dark gray, loop HEAT 8/9 yellow/pink) extend across the CRM1 dimer interface. (C) CRM1 is a Kap protein, characterized by its HEAT repeat architecture. It consists of 21 HEAT repeats that form a toroid-like structure (shades of gray). Each HEAT repeat is comprised of antiparallel helices (white circles), with A helices on the outer surface and B helices on the inner surface of the toroid. The schematic highlights that the known cargo-binding sites are distinct and non-overlapping: blue for Rev, yellow for the NES cleft, orange for SPN1, and pink for the RRE. White asterisks indicate the HEAT repeats (9 and 10) involved in CRM1 dimerization. (D) Rev (green) and SPN1 (purple; PDB:5DIS) interact with CRM1 at distinct, non-overlapping-binding sites (Rev site/blue; SPN1 site orange).

The asymmetry of Rev dimer binding is apparent in the way it docks to CRM1 outside the NES clefts. One Rev subunit is stacked across the CRM1 dimer and forms the predominant contacts, while the second Rev subunit remains docked underneath the complex via the Rev α-α interface. The interaction is more extensive on the CRM1 subunit bound to the RRE (CRM1_Rev/RRE_), highlighting the asymmetry observed throughout the structure. Most of the residues of CRM1 that interact with Rev are located on a loop between HEAT repeats 8 and 9 (residues 389–400), a region of CRM1 disordered in all previous structures of the human protein, including those with SPN1, whereas the loops in both subunits become structured in the NEC (Figures 3A and S5C).^44,45,64^ Although both loops are structured, their conformations differ slightly, likely due to variations in Rev engagement, with Rev interacting more extensively with the CRM1 subunit bound to the RRE. The charge of the electrostatic surface of the CRM1 dimer in this region diminishes when the loops become ordered, instead creating a hydrophobic platform for Rev binding (Figure S8).

Like Rev, the interaction between SPN1 and CRM1 involves both NES recognition and an external protein-binding site on the outside of the CRM1 toroid, in the case of SPN1 involving HEAT repeats 13 to 16 (Figure 3C).^43–45^ SPN1 is an adaptor protein that facilitates the nuclear import of spliceosomal small nuclear ribonucleoproteins (snRNPs) by binding the 2,2,7-trimethylguanosine (m3G) cap of snRNAs and interacting with the import receptor Importin-β.^65^ Once inside the nucleus, CRM1 mediates the export of SPN1 back to the cytoplasm, ensuring its recycling for subsequent rounds of snRNP import.^66^ The position of the NES within each protein most likely helps determine where the proteins dock on CRM1. The sites for Rev and SPN1 lie on opposite sides of the CRM1 NES cleft (Figure 3C). While the NES peptides in both cargos are bound in the same N-to-C orientation, SPN1 contains an N-terminal NES, whereas Rev has a C-terminal NES, resulting in the bulk of the protein being located on different ends of the NES (Figure 3D). To enable multipartite interactions with such different domain arrangements, CRM1 utilizes multiple protein-binding interfaces located on the outer surface of the HEAT repeats.^44,45,51–53,67^ This contrasts with all other Kap family members, which bind their cargos on the inner surface of their HEAT repeats.^35,36^

**Figure 4.**
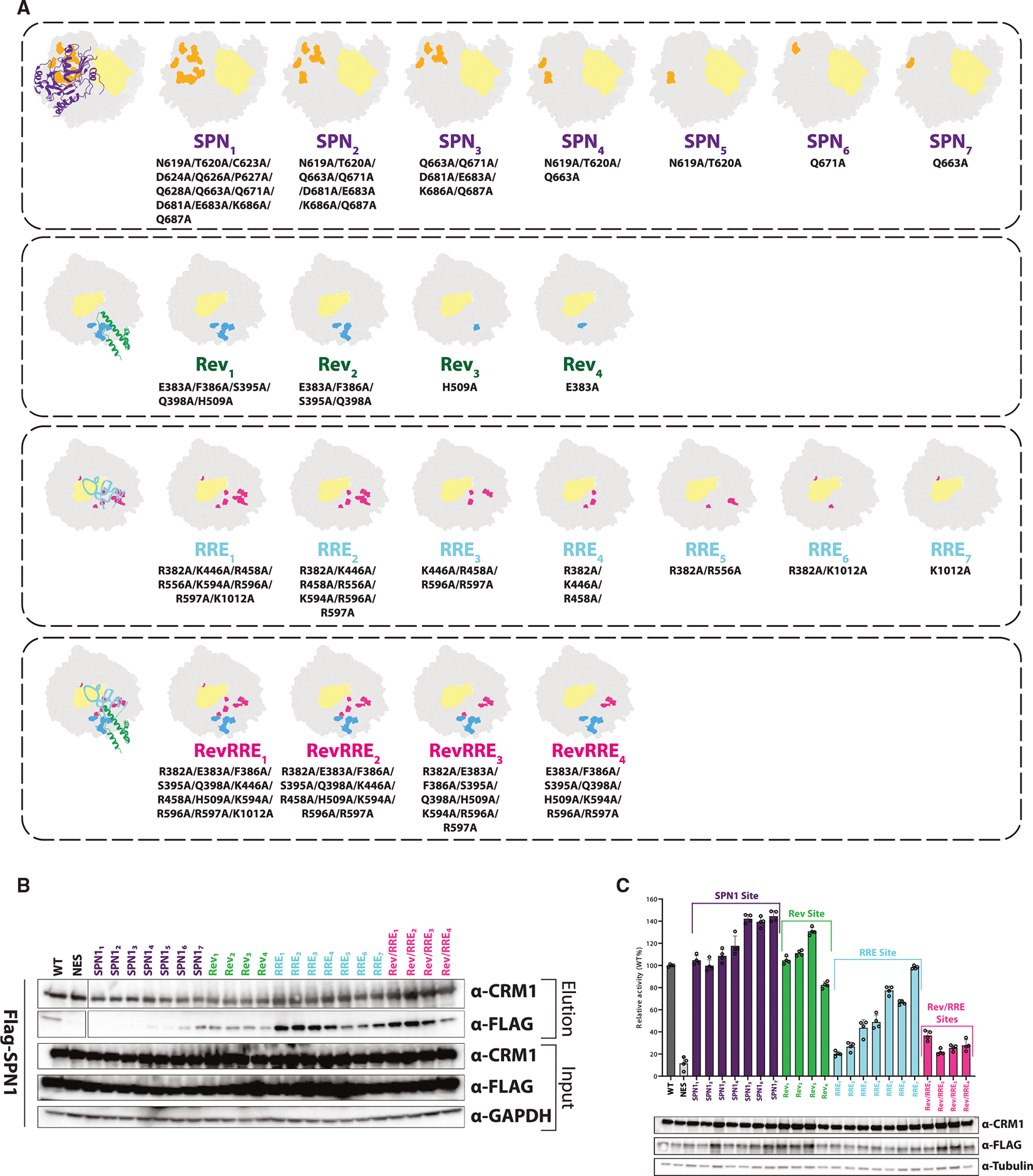
CRM1-binding sites are separate and distinct (A) The SPN1-, Rev-, and RRE-binding sites were mutated in various combinations. Each CRM1 construct is shown with the mutated residues highlighted and listed below, along with the corresponding name used in the subsequent assays. (B) To assess how these mutations affect SPN1 binding to CRM1, CRM1 was immunoprecipitated, and the samples were analyzed for SPN1 binding. (C) Rev nuclear export activity was measured using the same CRM1 variants in a dual-luciferase reporter assay. Data are presented as mean ± SEM, with individual data points shown. Non-relevant lanes were removed from the blot for clarity; only lanes used for quantification are shown. Uncropped blots are provided in Figure S14.

The presence of these distinct cargo-binding sites in CRM1, in addition to the NES recognition site, can help explain how CRM1 recognizes such a large inventory of cargos. To test whether the Rev- and SPN1-binding sites are functionally distinct, each site was mutated separately by substituting non-hydrophobic residues within the respective interfaces to alanine in various combinations (Figure 4A). Residues at the SPN1 interface were selected based on atomic contacts observed in a previously determined crystal structure.^43,45^ By contrast, the Rev-binding site was defined using our cryo-EM structure, which, while not allowing unambiguous identification of individual side-chain interactions, provided a clear framework to delineate the broader interaction surface. Guided by this, we selected buried, non-hydrophobic residues likely to contribute to binding for mutagenesis. The mutational tolerance of CRM1 in the NES-binding cleft, the RanBP1-binding site, and the Ran-binding site has been explored extensively^46,53,68,69^ but not around any other cargo-CRM1 interaction sites on the outer surface of the toroid. Each CRM1 variant we tested pulled down Ran to a similar extent in HEK293 cell immunoprecipitation (IP) experiments (data not shown), and each was analyzed by negative stain EM to ensure that the mutations did not affect the overall structure of CRM1 (Figure S9). Mutating 13 residues of the SPN1-binding site significantly impaired SPN1 binding (SPN1_1_; Figure 4B), while reducing the number of mutations sequentially restored SPN1 binding, with two single-point mutations fully restoring the CRM1 interaction (SPN1_6_ and SPN1_7_; Figure 4B). Unexpectedly, mutations in the Rev-binding site (Rev_1_–Rev_4_) actually enhanced SPN1 binding in most cases compared with wild-type CRM1, and similarly, mutations in the SPN1-binding site enhanced Rev-mediated nuclear export activity (Figure 4C). Interestingly, mutating the Rev protein-binding site in CRM1 had little effect on Rev activity. These results demonstrate that, beyond shared NES recognition, the Rev- and SPN1-binding sites on CRM1 are distinct, suggesting these regions could serve as targets for selective inhibition in future studies.

### Positioning of the two Rev NES peptides

**Figure 5.**
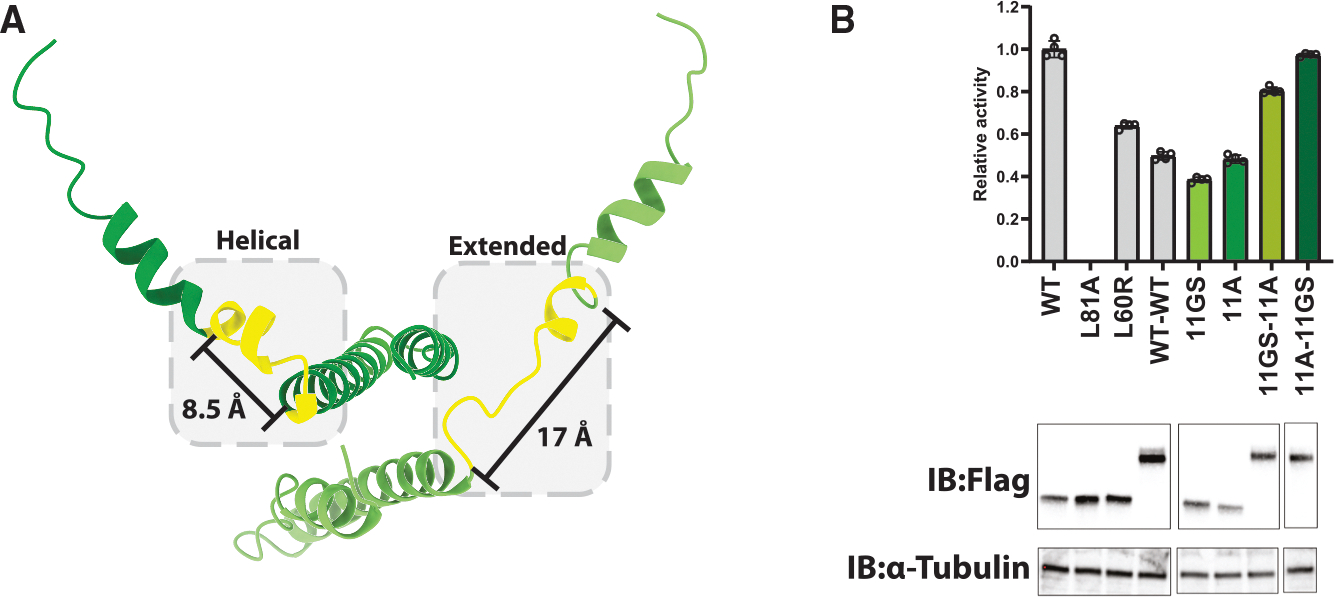
Structural asymmetry of the Rev ONL (A) Each Rev monomer inserts its NES into the NES cleft of its respective CRM1 subunit in the same orientation and manner. To accommodate the varying distances between Rev monomers while maintaining this conserved mode of NES engagement, the flexible OD-NES linkers (ONLs; yellow, residues 60–75) adopt different conformations within the Rev dimer. (B) Linked Rev dimers with ONLs engineered to adopt defined structures (11 alanines [11A] to promote a helical structure or 11 glycine-serine repeats [11GS] to create an extended conformation) were designed to assess ONL requirements. Constructs were tested for Rev nuclear export activity using a dual-luciferase reporter assay. Data are presented as mean ± SEM, with individual data points shown. Non-relevant lanes were removed from the blot for clarity; only lanes used for quantification are shown. Uncropped blots are provided in Figure S14.

One hallmark of CRM1-cargo recognition is the docking of an NES peptide into an NES-binding cleft in CRM1.^35,36^ Like monomeric SPN1, each of the two Rev NESs interacts in a well-characterized manner with the clefts formed by HEAT repeats 11 and 12,^44,47^ but the dimeric nature of the Rev complex reveals how the two Rev subunits position each NES. Because the Rev dimer is arranged in a stacked configuration, each NES requires a different distance and positioning to align properly with its respective CRM1 NES cleft. A striking asymmetry was immediately apparent, as suggested by previous biochemical studies,^26^ with the structurally well-defined linkers between the OD and NES (OD-NES-linker [ONL]) playing a key role (Figure S5B).^70^ These linkers adjacent to the NESs adopt markedly distinct conformations (Figure 5A), where the upper subunit ONL is helical and projects upward toward the β-face of the Rev hairpin, while the lower ONL rotates 180° and extends to reach the α-face of the hairpin. This linker flexibility allows both NESs to align in the two clefts in the same N-C orientation, compensating for the mirrored arrangement of the Rev dimer. This is consistent with previous experiments showing that substituting the linker with alanines or with a Gly-Ser repeat, which affects the helical propensities of both Rev subunits, reduced nuclear export and replication activities.^70^ To validate this asymmetry, we generated linked Rev dimers in which the N-terminal Rev molecule contained a helical 11-alanine (11A) ONL and the C-terminal Rev a flexible 11-Gly-Ser (11GS) ONL, or the converse orientation. Both linked dimers showed increased nuclear export activity compared with monomeric Rev containing either the 11A or 11GS substitutions (Figure 5B), and notably the dimer containing the N-terminal Rev with high helical propensity of the ONL (11A) and a C-terminal Rev with extended/flexible propensity of the ONL (11GS) was as active as wild-type Rev. By contrast, a linked dimer with wild-type ONLs exhibited low export activity, as expected given that Rev does not tolerate additions to its N or C termini.^70^ Negative stain 2D class averages of the wild-type linked dimer and the 11A/11GS linked dimer correlated with the nuclear export activity, where the wild-type linked dimer exhibited a mixture of monomeric and dimeric forms of CRM1, while the 11A/11GS yielded fully dimeric complexes (Figure S7). These results further validate the orientation of the Rev dimer in our model. These experiments suggest that the dimeric nature of Rev gives rise to two NESs and that enhancing Rev’s ability to optimally position these NESs correlates with increased nuclear export activity.

### RNA binding to one CRM1/Ran complex

**Figure 6.**
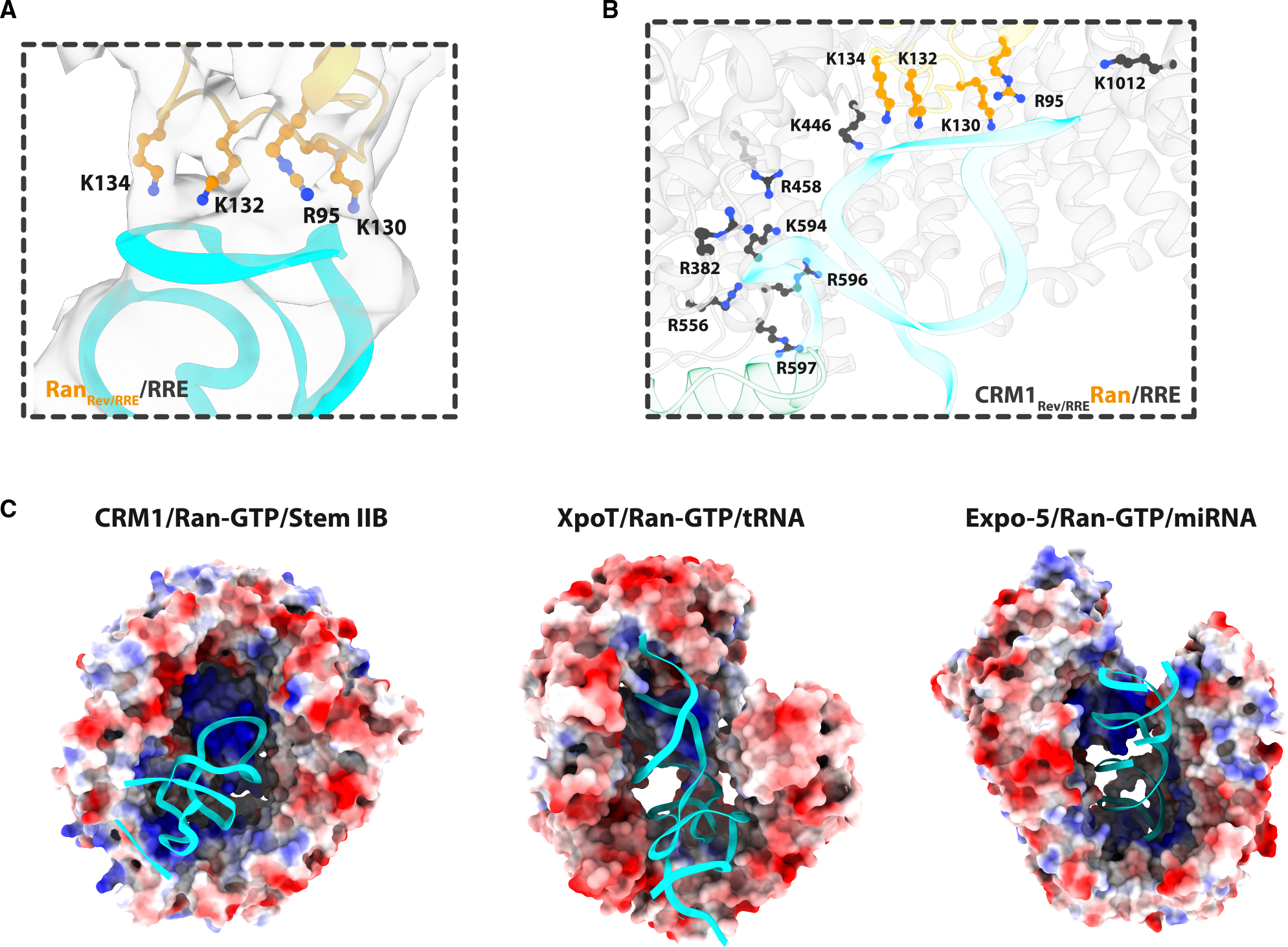
Rev mimics a conserved RNA-binding mechanism utilized by RNA trafficking exportins (A) The residues that form the RNA-binding pocket of CRM1 span the inner surface of the toroid, aligning with the angle at which the RRE interacts with both Rev and CRM1/Ran. (B) Four Ran residues—Arg95, Lys130, Lys132, and Lys134 (shown in orange)—contact the top of stem IIB, with clear density visible for their entire side chains. (C) This RNA-binding mode of CRM1 is similar to that of other exportins, where Ran serves as a cap, contributing to RNA specificity by influencing its length and wrapping around the RNA. Notably, the residues of Ran that interact with the RRE are the same ones that engage RNA in the crystal structures of XpoT (PDB: 3ICQ) and Expo-5 (PDB: 3A6P).

The NEC structure reveals an unexpected RNA-protein interface between CRM1/Ran and RRE stem IIB, in addition to the RevRNA interaction. While Rev is the critical adaptor protein for HIV RNA export, it is the RRE that strikingly interacts with every protein in the NEC. The IIB stem within the intact RRE is longer than in the co-crystal structure^20^ and is precisely the length to fit into the NEC reconstruction. The electrostatic surfaces of CRM1 and Ran show a positively charged pocket where the RRE is cradled between Rev and CRM1/Ran (Figure 6A). Ran contains two loops (91–95, 122–135) that cap the pocket and thus limit the length of RNA that can be accommodated. Four residues in Ran (Arg95, Lys130, Lys132, and Lys134) show clear density connected to the RRE (Figure 6B). Three of these residues (Arg95, Lys132, and Lys134) are the same residues that interact with the RNA cargos of Expo-5 and XpoT,^71,72^ illustrating viral mimicry of other Kap proteins^35,36^ (Figure 6C). XpoT and Expo-5 transport tRNAs or microRNA (miRNAs) by wrapping around the RNA, but, unlike CRM1, neither requires an adaptor protein.^71,72^ Based on the geometry of Rev’s interaction with the CRM1 dimer and stem IIB—and the way the RRE inserts into the composite pocket formed by CRM1 and Ran-GTP—we selected positively charged CRM1 residues that align with the angle of stem IIB for mutagenesis to test the importance of the RRE-binding site in CRM1 (Figures 4A and 6A). We then measured the effects of these mutations on Rev-mediated export activity.

Mutating 8 residues simultaneously to alanine (RRE_1_) decreased Rev activity to a level similar to a triple mutant in the NES cleft (NES^68^) and significantly more than mutating 5 residues within the Rev protein-binding site (Rev_1_), while simultaneously mutating both the RRE and Rev sites (Rev/RRE_1_–Rev/RRE_4_) decreased Rev activity further (Figure 4C). Thus, the RRE-binding site is a major contributor to CRM1 recognition. The additional RNA interaction increases the stability of the complex but alone cannot drive exportation. Reducing the number of RRE site mutations (RRE_1_-RRE_7_) sequentially restored Rev export activity, similar to the restoration of SPN1 binding with sequentially reduced mutations in the SPN1 protein site (SPN1_1_–SPN1_7_) (Figure 4B). Consistent with the importance of the RRE site for NEC formation, negative stain EM of CRM1 mutants in the RRE or Rev-binding sites (Rev_1_, RRE_1_, and Rev/RRE_1_) showed significantly reduced dimer formation, whereas the NES cleft mutation completely abolished the dimer (Figure S9). The NES cleft mutation also abolished SPN1 binding,^68^ and thus the RRE-, Rev-, or SPN1-CRM1 interactions alone are insufficient for complex formation without NES binding. These results emphasize the importance of the protein-protein interaction in the SPN1 complex and the RNA-CRM1 interaction in the HIV NEC and highlight the adaptability of CRM1 to both protein and RNP cargos.

### Extended architecture of the RRE

The structure of the NEC shows many well-resolved aspects of the dimeric core and the Rev/stem IIB complex, but the data do not allow a complete structural alignment of the RRE, suggesting that the RNA is highly flexible. To address the overall structure of the RRE, we obtained a low-resolution cryo-EM reconstruction of the Rev/RRE_355_ RNP without CRM1 (Figures S10 and S13; Table 2). While the intrinsic flexibility of the RNA made it difficult to align individual particles and identify most secondary structural features, it is clear that the RRE adopts an elongated conformation, unlike the more compact A-shaped structure suggested by a SAXS model.^30^ To attempt to model individual stem-loops into the EM reconstruction, we generated AlphaFold 3D^73^ predictions for RRE_355_, which overall resembled the elongated shape of the reconstruction (Figure S10), albeit with low confidence scores. We attempted rigid-body fitting of the AlphaFold model into the map and could not account for ~140 nucleotides (~40%) of the RRE, nor could we account for the additional Rev molecules in the complex, despite being crosslinked. However, one region of the map exhibited clear double-stranded RNA characteristics consistent in size and helical pitch with stem IIB (Figure S10). This view of the elongated nature of the RRE is consistent with the hypothesis that Rev molecules bind in a distributed manner throughout the structure, likely not associating beyond Rev dimers.

### Identification of other CRM1 recognition sites

**Figure 7.**
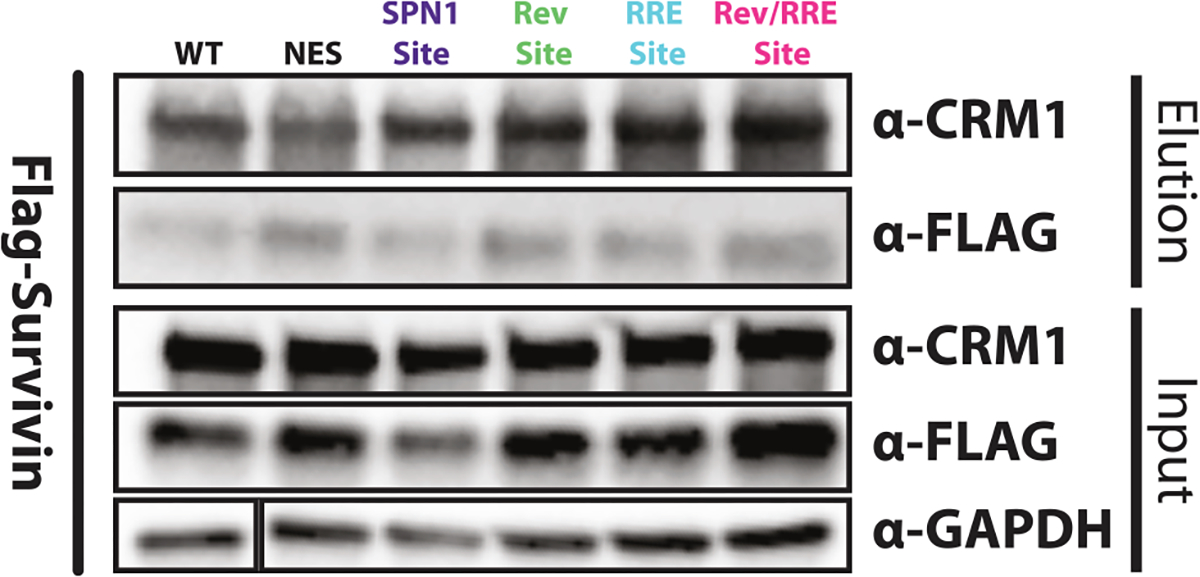
Survivin binds to a different CRM1 site Using the CRM1 variants from this study, CRM1 was immunoprecipitated, and the samples were analyzed for Survivin binding. Uncropped blots are provided in Figure S14.

To further explore the diversity of CRM1-binding sites for cargo recognition, we examined the binding of Survivin, a host cargo, to our panel of CRM1 mutants. Survivin is a member of the chromosome passenger complex (CPC) along with the proteins Aurora B, Borealin, and inner centromere protein (INCENP).^74^ The CPC is a highly conserved complex that is responsible for the movement and segregation of chromosomes during meiosis and mitosis.^75^ The ability of the CPC to target the centromeres of chromosomes is dependent on CRM1 and the NES of Survivin.^76^ In fact, CRM1 was first identified in yeast as a temperature-sensitive mutant that led to chromosomal disorganization, thus resulting in its original name as chromosomal region maintenance 1 (CRM1).^77^ To assess whether Survivin utilizes any of the three CRM1-binding sites identified in this study, we conducted Survivin pull-down experiments with CRM1 mutants (SPN1_1_, Rev_1_, RRE_1_, and Rev/RRE_1_) and found that each variant similarly pulled down Survivin (Figure 7), including the NES cleft mutant. Thus, Survivin apparently binds to another yet unidentified site, and its NES engages CRM1 in a manner that is mechanistically distinct from SPN1 and Rev.^50^ As Survivin is part of the CPC, which also includes Aurora B, known to interact with CRM1,^37^ its binding may be mediated via Aurora B. Additional studies are needed to identify the Survivin-binding site and to understand how the CPC interfaces with CRM1.

## DISCUSSION

### Redefining the Rev oligomer

Over four decades, structural studies of the Rev/RRE RNP have revealed important features of portions of the complex. The current view of Rev oligomerization suggests that Rev-Rev interactions beyond the core Rev dimer are required for NEC assembly. Size exclusion chromatography confirms the presence of multiple Rev molecules (Figure S8B), which are required to assemble a complete NEC.^54^ However, except for the core Rev dimer that directly interacts with CRM1, no other Rev dimers are seen in the final 3D reconstruction, suggesting that other Rev dimers do not interact with the core Rev dimer. Interestingly, residues in the ARM that engage the stem IA site are located on the opposite side of the ARM helix from those that bind stem IIB.^22^ This suggests that Rev dimers bound at this site adopt a distinct binding mode from the dimer at the stem IIB site, likely mediated by the β-β interface, allowing for these residues to be accessible for RNA interactions, albeit with 30–40-fold weaker affinity.^22,78^ Together, our data suggest a model in which multiple Rev dimers bind to the RRE in a scattered way but do not interact with each other. While they do not interact directly, their collective binding stabilizes the elongated RRE conformation and facilitates assembly of the complete NEC. This model may explain why mutations to the β-face disrupt Rev assembly on the RRE and why CRM1 does not bind Rev molecules at alternative RRE sites, effectively restricting CRM1 recruitment to the Rev dimer at stem IIB. Thus, the β-face appears to function primarily as a CRM1-interacting surface, and the so-called “OD” is not involved in classical oligomerization but instead serves as a dimerization and export interface. If the β-face is indeed critical for forming Rev dimers at additional RRE sites, it may not be possible to generate mutants that disrupt binding to these sites that do not simultaneously cripple the interaction with CRM1, as the interactions would be mutually exclusive. Nonetheless, the increased nuclear export activity observed with longer RREs containing additional weaker binding sites suggests that other facets of the Rev/RRE complex contribute to NEC formation and function.^15^

### The two NESs of the Rev dimer drive CRM1 dimerization

Our studies demonstrate that the binding of two NES peptides from the Rev dimer largely drives the dimeric form of CRM1. The CRM1 dimer interface, in turn, forms a docking site for the core of the Rev/RRE RNP, utilizing evolved species-specific residues.^54,60,61^ It is not yet known if CRM1 dimerization is used in other viral or host cargo complexes, but the sequence diversity of the interface is intriguing and may suggest a broader role for the CRM1 dimer beyond the HIV NEC. The CRM1 dimer interface utilized by Rev may be used by host cargos as well and may not be restricted to dimeric cargos but also monomers or higher-order oligomers. The Rev dimer subunit arrangement in the NEC, dictated by the bound RNA, coupled with the ability of the Rev ONL to adopt different ordered conformations, orients the two NES peptides to grasp the two NES-binding clefts in the CRM1 dimer. NES peptide binding appears to dominate NEC assembly, as mutating the NES cleft completely abolishes NEC assembly, whereas mutating both the RRE- and Rev-binding sites still allows a small population of the NEC to form (Figure S9). In comparison, the CRM1-SPN1 complex is monomeric, and mutating either the NES cleft or the SPN1-binding site abolishes complex formation (Figure S9). Thus, in addition to NES binding, the relative importance of a binding site on the outside of the CRM1 toroid may differ for monomeric or dimeric cargos. The HIV NEC structure begins to establish some principles for CRM1 recognition of oligomeric proteins or RNPs, many of which are found among the >1,000 cargos identified to date.^37,38,79^

### RNA is integral to RNP cargo recognition

One striking feature of the NEC structure is the rather extensive interface between CRM1/Ran and the Rev/RRE cargo, burying 3,456 Å of surface, including 1,872 Å from the two essential NES interactions and 512 Å from the RNA. Thus, cargo recognition in the NEC is a composite of NES binding, Rev protein-binding to the outside of the toroid, and RNA binding to the inside. Indeed, the importance of the RNA interaction is demonstrated by mutation of the RNA-CRM1 interface, which greatly weakens NEC formation, whereas mutation of the Rev-CRM1 interaction has little effect (Figure 4C). A previous study hinted at a direct interaction between the RRE and CRM1/Ran, whereby an RRE structural variant was found that rescued activity of a *trans*-dominant negative form of Rev (RevM10) containing two mutations in the NES.^55,80^

The asymmetric binding of the RRE to just one subunit of the CRM1/Ran dimer, despite having two identical RNA-binding pockets, further illustrates how the two NESs of the Rev dimer provide the major driving force for CRM1 dimer formation. The presence of such an obvious RNA-binding site in CRM1 may also be expected to facilitate interactions of other RNP cargos, including monomeric cargos. The electrostatic nature of the composite CRM1/Ran RNA-binding site and the conservation of binding modes with other RNA exportins, Expo-5^72^ and Xpo-T^71^ (Figure 6C), is yet another demonstration of the ability of a virus to mimic host interactions. CRM1 is the only exportin known to date to export RNPs^42^ and thus appears to be a highly adaptable Kap, which uses multipartite binding to interact with both components of its RNP cargo, binding protein on the outside and RNA on the inside. The requirement for NES binding, in addition to RNA binding, explains why CRM1 cargos include proteins and not RNA alone.

### Multipartite recognition of protein and RNP cargos

Understanding how CRM1 is able to recognize such a large and diverse repertoire of proteins and RNP complexes has long been a subject of inquiry.^35,36^ NES peptide recognition is one critical component and has led to focused efforts to inhibit specific cargo interactions.^81–83^ It is well-established that CRM1 binds regulatory proteins at distinct sites that control cargo loading and release,^45,51,52,84,85^ but less is known about other sites used for cargo recognition. The discovery of two additional protein-binding sites and an RNA site described here highlights the importance of multipartite interactions in addition to NES binding. In the HIV NEC, the CRM1-RRE interaction ensures that Rev is not exported until it is bound to the RRE, showing another advantage of multipartite recognition. This quality control concept aligns with recognition of SPN1, where CRM1 interactions with the m_3_G cap-binding domain ensure that SPN1 is not exported until it has released the U snRNPs in the nucleus.^66^

The ability to utilize different binding surfaces of CRM1 allows it to adapt to a wide variety of cargos with distinct molecular characteristics, including RNAs. The finding that Survivin appears to use another binding site different from the three identified sites and even binds CRM1 when the NES cleft is mutated (Figure 7) suggests that export complexes can be assembled through numerous surfaces, potentially involving additional proteins such as those of the CPC. The mechanisms of CRM1-cargo recognition undoubtedly will expand further as more cargos are identified beyond the already substantial list.^37–39,41,79,86^ These are likely to uncover additional protein docking sites around the toroid, positioned by NES peptide docking, protein architecture, size, and flexibility. Multipartite recognition allows for compensating interactions over a wide range of NES affinities,^48,49^ and it may be possible for CRM1 to recognize cargos even without an NES peptide. A firmer structural understanding of cargo recognition features, such as that provided by the HIV NEC structure, may facilitate the development of specific CRM1 inhibitors.

### Limitations of the study

While our study elucidates structural and functional impacts of the different interfaces critical to the HIV-1 NEC, including how Rev engages CRM1 and the distinct cargo-binding sites of CRM1, several limitations remain. We performed our analyses primarily in HEK293T cells, with some experiments in BHK21 cells, and the effects of disrupting these interfaces on Rev and CRM1 during full viral replication in primary cells or *in vivo* remain to be validated. Additionally, the components of the NEC were modified for structural analysis and may not fully represent the native forms and conditions present during HIV replication. Our cryo-EM analysis is based on a single Rev-RRE-CRM1/Ran-GTP complex. However, the extensive data collected and challenges in achieving a high-resolution reconstruction highlight the dynamic and flexible nature of this complex, which is not fully captured in the presented structure. Future work using *in vivo* infection models and additional biochemical assays will be important to fully understand the mechanistic consequences of the CRM1 interfaces utilized during HIV replication.

### RESOURCE AVAILABILITY

#### Lead contact

Further information should be directed to and will be fulfilled by the lead contact, Alan D. Frankel (frankel@cgl.ucsf.edu).

#### Materials availability

All unique reagents generated in this study are available from the lead contact upon execution of a materials transfer agreement.

#### Data and code availability

- All data generated or analyzed during this study are included in this published article and its supplemental information. The cryo-EM density map for the NEC has been deposited in the Electron Microscopy Data Bank under accession code EMDB-42494, and the coordinates have been deposited in the Protein Data Bank under accession PDB:8URJ. The cryo-EM density map for the RRE has been deposited in the Electron Microscopy Data Bank under accession code EMDB-70347. They are publicly available as of the date of publication.
- This paper does not report original code.
- Any additional information required to reanalyze the data reported in this paper is available from the lead contact upon request.

## STAR★METHODS

### METHOD DETAILS

#### Expression and protein purification

##### Expression and purification of wild-type CRM1

Full-length, codon optimized human CRM1 was cloned into a modified pFastBac vector containing a mammalian cytomegalovirus (CMV) promoter between the BamHI and NotI restriction sites.^101–103^ The construct was assembled using G-blocks (IDT, US) and Gibson Assembly (Table S1; New England Biosciences, US). CRM1 has an N-terminal octa-histidine tag followed by the maltose binding protein (MBP) separated by a tobacco etch virus (TEV) protease site. This plasmid was transformed into the DH10Bac *Escherichia coli* strain to generate the recombinant bacmid DNA which was then transfected into insect cells to generate the BacMam virus.^104^ CRM1 was expressed in FreeStyle HEK293-F cells suspended in FreeStyle 293 Expression Medium (Invitrogen) supplemented with 2% (v/v) fetal bovine serum (FBS). Cells were grown at 37°C and 8% CO_2_ on an orbital shaker to a cell density of ~2.0 × 10^6^ per mL at which point they were transduced with P3 BacMam virus and expression was enhanced by the addition of sodium butyrate to a final concentration of 10 mM. Cells were harvested 30 hours post-transduction and snap-frozen in liquid nitrogen and stored at −80°C until purification. CRM1 was resuspended in 5 mL of lysis buffer (20 mM Tris pH 7.5, 600 mM NaCl_2_, 2 mM magnesium acetate (MgOAc), 2 mM β- mercaptoethanol (BME), 10 % glycerol (v/v) and 1 mM guanosine diphosphate (GDP)) for each gram of cell pellet. Lysis buffer was supplemented with Halt^™^ Protease Inhibitor (Thermo Scientific) and phenylmethylsulfonyl fluoride (PMSF) to a final concentration of 1 mM. CRM1 was purified by batch binding to amylose resin (New England Biosciences, US) overnight at 4°C. The amylose resin was washed in Buffer A (20 mM Tris pH 7.5, 600 mM NaCl_2_, 2 mM MgOAc, 2 mM BME, 20 % glycerol (v/v) and 20 mM imidazole pH 7.5) followed by elution in Buffer A supplemented with 10 mM maltose. The eluted CRM1 was cleaved with TEV protease at room temperature and the protease and His_8_ – MBP tag were removed from CRM1 using Ni-NTA resin (Thermo Scientific, US). CRM1 was then exchanged into Buffer B (20 mM Tris pH 7.5, 100 mM NaCl_2_, 2 mM MgOAc, 2 mM BME and 20 % glycerol (v/v)) and loaded onto a Mono-Q 5/50 GL column (Cytivia, Sweden). CRM1 was eluted with a gradient of 0%–100% Buffer C (20 mM Tris pH 7.5, 1 M NaCl_2_, 2 mM MgOAc, 2 mM BME, 20 % glycerol (v/v)). Fractions were analyzed by SDS-PAGE, pure fractions were pooled, frozen in liquid nitrogen and stored at −80°C. CRM1 concentrations were determined by Bradford Assays using bovine serium albumin (BSA) standards (Thermo Scientific).

##### Expression and purification of binding-site variants of CRM1

Full-length human CRM1 with an N-terminal 2x Strep tag was cloned into the pcDNA 4/TO plasmid (Thermo Fisher Scientific, US) between the restriction sites NotI and XhoI. The CRM1 variants were generated by site directed mutagenesis (Table S2). The CRM1 variants were expressed using the Expi293 transient expression system (Thermo Fisher Scientific, US) using manufacturer supplied protocols. Cells were harvested 48 hours post-transfection and snap-frozen in liquid nitrogen and stored at −80°C until purification. CRM1 was resuspended in 5 mL of lysis buffer (20 mM Tris pH 7.5, 600 mM NaCl_2_, 2 mM MgOAc, 2 mM BME, 10 % glycerol (v/v) and 1 mM GDP) for each gram of cell pellet. Lysis buffer was supplemented with Halt^™^ protease Inhibitor and PMSF to a final concentration of 1 mM. CRM1 was purified by batch binding to Strep-Tactin XT 4Flow resin (IBA, Germany) overnight at 4°C. The Strep-Tactin resin was washed in Buffer D (20 mM Tris pH 7.5, 600 mM NaCl_2_, 2 mM MgOAc, 2 mM BME and 20 % glycerol (v/v)) followed by elution in Buffer D supplemented with 1x BXT Buffer (IBA, Germany). The eluted CRM1 was then exchanged into Buffer B and loaded onto a Mono-Q 5/50 GL column. CRM1 was eluted with a gradient of 0%–100% Buffer C. Fractions were analyzed by SDS-PAGE, pure fractions were pooled, frozen in liquid nitrogen and stored at −80°C. CRM1 concentration was determined by Bradford assays using BSA standards.

##### Expression and purification of Rev variants

Plasmids used for wild type Rev HIV-1 strain HXB3 and Rev L60R HIV-1 strain HXB3 have been previously described.^22^ Rev L81A HIV-1 strain HXB3 was generated by site directed mutagenesis (Table S2). Rev NS2Val was generated by modifying the previously described Rev construct by PCR with primers that amplified Rev on either side of the native NES adding 13 amino acids to both the N-terminal and C-terminal halves (Table S2). The two PCR products contained a 38 bp overlap aiding in the PCR amplification of the full Rev NS2Val construct which was then be cloned into the original plasmid at NdeI and XhoI restriction sites to replace wild-type Rev with the Rev NS2Val. This increased the *rev* gene from 116 amino acids to 121 amino acids. The above Rev variants were expressed as previously described.^22^ Cells were harvested and snap-frozen in liquid nitrogen and stored at −80°C until purification. Genes blocks of the Rev linked dimer variants were designed to have the same Rev DNA sequence as in Rev wild-type plasmid, with the N-terminal Rev followed by a (GGGGS)_3_ linker and a codon-optimized DNA sequence for Rev in the C-terminal position to avoid direct DNA sequence repeats (Table S1). For the 11A/11GS construct, residues 62–72 were replaced with 11 alanines in the N-terminal Rev with (GGS)_3_GS in the C-terminal Rev. The gene fragments (IDT) were designed to have a 38 bp overlap at the 5′ and a 16 bp overlap at the 3′ and were cloned into the wildtype plasmid digested with NdeI and XhoI using In-Phusion Snap Assembly (Takara Bio USA). The linked dimer constructs were expressed in *E. coli* strain BL21/DE3. Cells were grown to OD_600_ = 0.6–0.8 at 37°C in 2XYT media (Fisher Scientific, US) with 100 μg/mL carbenicillin. Cells were then cooled to 18°C and isopropyl-β-D-thiogalactopyranoside (IPTG) was added to 1 mM to induce expression. Cells were harvested the following day and snap-frozen in liquid nitrogen and stored at −80°C until purification. For purification, cells were resuspended in 5 mL of lysis buffer (50 mM Tris pH 7.5, 250 mM NaCl_2_, 5 mM BME, 10 mM imidazole pH 7.5, 0.1% (v/v) Tween-20) per gram of cell pellet. Lysis buffer was supplemented with Halt^™^ protease Inhibitor and PMSF to a final concentration of 1 mM and lysozyme to a final concentration of 0.5 mg/mL. Endogenous RNA from *E. coli* was removed by adding solid NaCl_2_ (final concentration of 2 M) and urea (final concentration of 1M) to the cleared lysate before addition to Ni-NTA resin. Batch binding was carried out at 4°C for 1 hour. The Ni-NTA resin was washed with Buffer E (50 mM Tris pH 7.5, 2 M NaCl_2_, 5 mM BME, 10 mM imidazole pH 7.5, 0.1% (v/v) Tween-20, 1M Urea) and Buffer F (125 mM phosphate buffer pH 7.5, 250 mM NaCl_2_, 5 mM BME, 10 mM imidazole). Rev was eluted by stepwise chromatography in Buffer F (50–500 mM imidazole pH 7.5). Fractions were analyzed by SDS-PAGE, pure fractions were pooled, frozen in liquid nitrogen, and stored at −80°C. Rev concentration was determined by Bradford assays using a cysteine-free version of Rev as a standard. Before adding the RRE to generate complexes, Rev was dialyzed overnight in Buffer F (10 mM imidazole pH 7.5) supplemented with 10% (v/v) glycerol in the presence of TEV protease. The N-terminal hexa-histidine tag and GB1 solubility-enhancing domain and the N-terminal hexa-histidine tagged TEV protease were removed using Ni-NTA resin.

##### Ran Q69L 180 expression and purification

Human Ran Q69L 180 was generated by site-directed mutagenesis to add a stop codon in place of alanine 181 to the previously described Ran plasmid^54^ and was expressed as previously described (Table S2). Cells were harvested and snap-frozen in liquid nitrogen and stored at −80°C until purification. Ran Q69L 180 was resuspended in 5 mL of lysis buffer (64 mM Tris pH 7.5, 400 mM NaCl_2_, 5 mM MgCl_2_, 2 mM BME, 10% (v/v) glycerol) per gram of cell pellet. Lysis buffer was supplemented with Halt^™^ protease Inhibitor and PMSF to a final concentration of 1 mM and lysozyme to a final concentration of 0.5 mg/mL. RanQ69L 180 was first purified by stepwise elution from Ni-NTA in Buffer G (64 mM Tris pH 7.5, 400 mM NaCl_2_, 5 mM MgCl_2_, 2 mM BME, 10% (v/v) glycerol, 50–500 mM imidazole pH 7.5) and dialyzed overnight in Buffer G (10 mM imidazole pH 7.5) at 4°C in the presence of N-terminal hexahistidine tagged TEV protease. The TEV protease and the deca-histidine tag were removed with Ni-NTA resin. Ran Q69L 180 was concentrated with a 10 kD MWCO Amicon Centrifugal Filter at 3.5K × g to a final volume of 2.5 mL and exchanged into Low Salt Buffer H (25 mM HEPES pH 7.5, 100 mM KCl, 4 mM BME, 10% (v/v) glycerol, 30 μM GTP) using a PD-10 desalting column (Cytiva, Sweden). Cation exchange was performed on a Mono-S column 5/50 GL column (Cytivia, Sweden) with a linear gradient 0 %–100 % high Salt Buffer I (25 mM HEPES pH 7.5, 1 M KCl, 4 mM BME, 10% (v/v) glycerol, 30 μM GTP). Fractions were analyzed by SDS-PAGE, pure fractions were pooled, frozen in liquid nitrogen and stored at −80°C. RanQ69L 180 concentration was determined by Bradford assays with a BSA standards.

For *in vitro* assays, Ran Q69L 180 was subcloned from the pET19b expression plasmid into the pcDNA4/TO vector, adding an N-terminal HA tag, and inserted between the NotI and XhoI restriction sites (Table S2).

###### _MBP_Nup214_1916–2033_ FG peptide expression and purification.

_MBP_Nup214_1916–2033_ was cloned into pET22b between the NdeI and XhoI restriction sites using a synthetic gene fragment (Table S1; Twist Bioscience, US). This construct contains an N-terminal MBP tag followed by 118 residues of Nup214 (residues 1916–2033), leucine and glutamate residues (LE) and a C-terminal hexa-histidine tag. _MBP_Nup214_1916–2033_ was expressed in Rosetta^™^(DE3) pLysS cells. Cells were grown to OD_600_ = 0.6–0.8 at 37°C in 2XYT media with 100 μg/mL carbenicillin. Cells were then cooled to 16°C and expression was induced with IPTG to a final concentration of 0.5 mM. Cells were harvested after 20 hours and snap-frozen in liquid nitrogen and stored at −80°C until purification. One liter of _MBP_Nup214_1916–2033_ cell pellet was resuspended in 80 mL of lysis buffer (50 mM HEPES pH 7.4, 500 mM NaCl_2_, 5% (v/v) glycerol, 20 mM imidazole and 1 mM 1,4-dithiothreitol (DTT)). Cleared lysate was added to equilibrated Ni-NTA resin, sample was flowed over resin twice. The resin was then washed with a 100 mL of lysis buffer. _MBP_Nup214_1916–2033_ was eluted from the Ni-NTA resin with 20 mL of Buffer J (50 mM HEPES pH 7.4, 500 mM NaCl_2_, 5% (v/v) glycerol, 500 mM imidazole and 1 mM DTT). _MBP_Nup214_1916–2033_ was then added to amylose resin. Batch binding was carried out at 4°C rotating end over end for 1 hour. Resin was then washed with 60 mL of Buffer K (20 mM Tris pH 7.4, 200 mM NaCl_2_, 1 mM ethylenediaminetetraacetic acid (EDTA), 2 mM DTT). Protein was eluted with 20 mL of Buffer K supplemented with maltose to a final concentration of 10 mM. Fractions were analyzed by SDS-PAGE, pure fractions were pooled, frozen in liquid nitrogen and stored at −80°C. _MBP_Nup214_1916–2033_ concentration was determined by Bradford assays using BSA standards.

##### Expression and purification of Snurportin-1

Full-length human Snurportin-1 (SPN1) with an N-terminal FLAG tag was cloned into the pcDNA4/TO vector between the NotI and XhoI restriction sites using a synthetic gene fragment (Table S1, Twist Biosciences, US) for *in vitro* assays. For recombinant expression, SPN1 was subcloned from this plasmid using primers that replaced the N-terminal FLAG tag with a 2x Strep tag, while maintaining the same pcDNA4/TO backbone and cloning sites (Table S2). SPN1 was expressed using the Expi293 transient expression system using manufacturer supplied protocols. Cells were harvested 48 hours post-transfection and snap-frozen in liquid nitrogen and stored at −80°C until purification. SPN1 was resuspended in 5 mL of lysis buffer (50 mM Tris pH 7.5, 200 mM NaCl_2_, 2 mM DTT, 2 mM MgOAc and 1 mM EDTA) for each gram of cell pellet. Lysis buffer was supplemented with Halt^™^ protease Inhibitor and PMSF to a final concentration of 1 mM. SPN1 was purified by batch binding to Strep-Tactin XT 4Flow resin overnight at 4°C. The Strep-Tactin resin was washed in lysis buffer followed by elution in lysis buffer supplemented with 1x BXT buffer. The eluted SPN1 was then exchanged into Buffer L (50 mM Tris pH 7.5, 50 mM NaCl_2_, 2 mM DTT and 2 mM MgOAc) using a PD-10 desalting column and loaded onto a Mono-Q 5/50 GL column. SPN1 was eluted with a gradient of 0%–100% Buffer M (50 mM Tris pH 7.5, 1 M NaCl_2_, 2 mM DTT and 2 mM MgOAc). Fractions were analyzed by SDS-PAGE, pure fractions were pooled, frozen in liquid nitrogen and stored at −80°C. SPN1 concentration was determined by Bradford Assay with a BSA standard.

##### RNA transcription and purification

The plasmid used to express RRE_234_ (HIV-1 strain SF-2) has been previously described^54^ and was modified by site directed mutagenesis to express RRE_234(61)_ containing the G262A and G269A mutations^55^ (Figure S2; Table S2). To express RRE_355_ (HIV-1 strain SF-2) a gene block (IDT, US) with SacII and EcoRI restriction sites at the 5′ end and XbaI at the 3′ end replaced RRE_234_ in the same vector. Plasmids were linearized utilizing the EcoRI site and RNA was transcribed by T7 polymerase followed by purification by denaturing urea-PAGE.^22,54^ The pure RNA pellets were resuspended in 0.5 mM HEPES-KOH pH 7.5. RNA was annealed by heating to 95–100°C then flash cooled on ice. RNA was quantified by UV spectroscopy using a 260 nm extinction coefficient of 2.5 × 10^6^ for RRE_234_ and RRE_234(61)_ and a 260 nm extinction coefficient of 4.3 × 10^6^ for RRE_355_.

##### Nuclear export complex assembly

Rev and RRE were mixed in an 8:1 ratio for the monomer proteins and 4:1 ratio for the linked dimers. The RNP mixtures were loaded onto a Superdex 200 Increase 10/300 column (Cytiva, Sweden) and purified in Buffer N (50 mM HEPES pH 7.5, 50 mM KCl, 2 mM BME, 2% (v/v) glycerol). The RNPs were then mixed with RanQ69L 180, CRM1 and _MBP_Nup214_1916–2033_ in a ratio of 1:8:4:8 in Buffer O (50 mM HEPES pH 7.5, 100 mM KCl, 2 mM MgOAc, 2 mM BME, 30 μM GTP). The complex was then incubated at 37°C for 15 minutes and kept at room temperature until it was added to EM grids. Complexes assembled for negative stain only did not contain _MBP_Nup214_1916–2033_. The complex was quantified by UV spectroscopy using a 260 nm extinction coefficient of 2.5 × 10^6^ for RRE_234_ and RRE_61_ and a 260 nm extinction coefficient of 4.3 × 10^6^ for RRE_355_.

##### GraFix-crosslinked RNP preparation

Following size-exclusion chromatography (SEC) purification, Rev/RRE_355_ RNP complexes were crosslinked using the GraFix^105,106^ method with 0.15% glutaraldehyde (GTA) or 0.1% formaldehyde (FA). The purified complexes (~6 uM) were ultracentrifuged through a 5% to 25% glycerol gradient containing either GTA or FA, enabling both purification and gradual fixation. Fractions were collected and analyzed via SDS-PAGE and silver staining and those displaying multiple bands, with each band corresponding to approximately two Rev monomers, were selected to ensure proper crosslinking without over-fixation. These fractions were pooled, concentrated, and buffer exchanged in Buffer P (50 mM HEPES pH 7.5, 50 mM KCl, 2 mM BME, 2.5% glycerol). The final complex concentration was determined by UV spectroscopy, using a 260 nm extinction coefficient of 4.3 × 10^6^.

##### Negative-stain electron microscopy

EM grids of negatively stained CRM1 alone, NEC, CRM1/SPN1, or GraFix-crosslinked RNP complexes were prepared using established protocols.^107^ Briefly, 3 uL of sample at ~0.010–0.012 mg/mL for CRM1 complexes and 0.025 mg/mL for RNP complexes was applied to a glow-discharged copper grid layered with amorphous carbon (Ted Pella Inc., US). After 30 seconds the sample was blotted off with filter paper (Whitman, #1) and washed twice using 3 μL of 2% (w/v) uranyl formate and blotted. Negatively stained grids were imaged on either a FEI Tecnai T12 electron microscope (Thermo Fisher Scientific, US) equipped with a LaB6 filament and a 4K CCD camera (UltraScan 895, Gatan, Inc.), operated at 120 kV, or a FEI Tecnai T20 electron microscope (Thermo Fisher Scientific, US) equipped with a LaB6 filament and a TVIPS TemCam F816 (8K x 8K) scintillator-based CMOS camera (TVIPS, Germany), operated at 200 kV, or a Talos L120C (Thermo Fisher Scientific, US) equipped with a LaB6 filament and a Ceta-D camera (Thermo Fisher Scientific, US) operated at 120 kV. Particle picking and 2D classification was carried out using RELION.^90,91^

##### Cryo-EM grid preparation

###### NEC grid preparation.

Functionalized graphene oxide (GO) grids^108,109^ were used to mitigate complex disassociation and the preferred orientations as observed with non-modified grids or simply GO alone treated grids. Functionalized GO grids were prepared^110^ using a variety of single-stranded DNA (ssDNA) oligos of various lengths and sequences or using polyamine (Table S3).^111,112^ Briefly, first GO was applied to 300 mesh 1.2/1.3 R Au Quantifoil grids (Qunatifoil, Germany) that were plasma cleaned in argon gas for 10 seconds using an established protocol.^109^ To functionalize GO grids with polyamine, 5 μL of ethylenediamine was added to 5 mL of dimethyl sulfoxide (DMSO) and grids were incubated in this solution for ~ 3 hours. The ethylenediamine/DMSO mixture was aspirated off and the grids were then incubated for 1 minute in 5 mL of DMSO followed by aspiration. Next the grids were incubated for 1 minute in deionized water (ddH_2_O), three times and then incubated in ethanol for 1 minute three times. Grids were air dried before either being used or were stored at −20°C. For ssDNA functionalization, DNA oligos were first resuspended in DMSO to a final concentration of 200 μM. Single grids were floated GO side down on 20 μL of ss-DNA/DMSO solution in a 1.5 mL Eppendorf tube and tubes were placed in an Eppendorf thermomixer (Eppendorf, US) at 25°C overnight. The following day the grids were rinsed with 200 μL of ddH_2_O followed by 200 μL of ethanol. Grids were air dried before being used or were stored at −20°C. Three μL of sample at ~0.5–0.7 mg/mL was added to the functionalized grids and incubated for 1 minute before being side blotted off outside of the vitrobot, using two applications of 3 μL of Buffer J for washing. After adding the final buffer drop, the grid and the tweezers were placed in the vitrobot for immediate blotting and plunging. Grids were frozen using a FEI Vitrobot Mark IV (Thermo Fisher Scientific, US) with a blotting time of 1 second. Vitrobot was set to 100% humidity and 22°C. Grids were plunge-frozen in liquid ethane and cooled by liquid nitrogen.

###### RNP grid preparation.

Three μL of sample at ~0.15–0.2 mg/mL was added to 300 mesh 1.2/1.3 R Au Quantifoil grids (Qunatifoil, Germany). Grids for the 0.1% FA samples were glow-discharged for 40 seconds at 15 mA prior to sample application and freezing. Grids for the 0.15% GTA were not glow-discharged. Grids were frozen using a FEI Vitrobot Mark IV (Thermo Fisher Scientific, US) with a blotting time of 6 seconds. Vitrobot was set to 100% humidity and 10°C. Grids were plunge-frozen in liquid ethane and cooled by liquid nitrogen.

#### Cryo-EM data acquisition

##### NEC data acquisition

Data sets were acquired on a Titan Krios (Thermo Fisher Scientific, US), operated at 300 kV and equipped with a K3 direct electron detector and a BioQuantum energy filter (Gatan, US) with a slit set to 20 eV. A defocus range of 0.8 to 2 μm under focus was used. Super-resolution movies were collected semiautomatically using SerialEM^87^ and recorded with a super-resolution pixel size of 0.417 Å/pixel for the ssDNA-GO grids and 0.4175 Å /pixel for the polyamine-GO grids. A nominal magnification of 105kx was used for all samples, a total dose of 66 e−/Å^2^ over 115 frames was used for the ssDNA samples and a total dose of 68 e−/Å^2^ over 118 frames was used for the polyamine-GO samples. Of the 22,762 movies that were recorded for the polyamine-GO grids, 7,720 movies were collected at 0° tilt, 11,190 movies were collected at 15° tilt and 3,852 movies were collected at 30° tilt. Of the 24,447 movies that were recorded for the ssDNA-GO grids, 7,286 movies were collected at 25° tilt. Movies were motion corrected using MotionCor2^88^ and binned 2 × 2 by Fourier cropping to a final pixel size of 0.834 Å per pixel for ssDNA samples and 0.835 Å per pixel for polyamine grids.

##### RNP data acquisition

Data sets were acquired on a Glacios Arctica (Thermo Fisher Scientific, US), operated at 200 kV and equipped with a K2 direct electron detector (Gatan, US). A defocus range of 0.8 to 2 μm under focus was used. Super-resolution movies were collected semiautomatically using SerialEM and recorded with a super-resolution pixel size of 0.575 Å/pixel.^87^ A nominal magnification of 36kx was used for all samples, a total dose of 95 e−/Å^2^ over 60 frames. Movies were motion corrected using MotionCor2 and binned 2 × 2 by Fourier cropping to a final pixel size of 1.1505 Å per pixel.

#### Image processing

##### NEC image processing

Initial processing for the ssDNA samples was performed in CryoSPARC.^89^ Dose-weighted motion corrected sums for the ssDNA samples were used for the estimation of the contrast transfer function (CTF) utilizing Patch CTF in CryoSPARC. Particles were auto picked individually for all six of the ssDNA samples using a Gaussian template in CryoSPARC. Particles were extracted with a box size of 440 pixels and binned to 220 pixels. 2D classification was carried out in CryoSPARC. Particles from the different collections were combined and 2D class averages were used to re-picked particles by the template-based method in CryoSPARC. 2D classification was ran multiple times to remove particle images that did not resemble the NEC. Particle files were converted to RELION using UCSF pyEM^93^ for subsequent image processing. Image processing for the polyamine samples was performed in RELION.^91^ Dose-weighted motion corrected sums were used for the estimation of the CTF utilizing CTFFIND4 wrapped within the RELION graphical user interface.^92^ Particles were picked using a 3D template from the combined results from the ssDNA samples. Particles were extracted with a box size of 440 pixels and binned to 110 for 2D classification. After multiple rounds of 2D classification, selected particles were re-extracted in RELION and binned to 220 pixels. Particles from 2D classification from the different functionalized grids and tilt angles were combined and 3D classification and refinement were carried out in RELION. Employing symmetry expansion and 3D variability methods^113^ led to a subset of particles with a sufficient sampling of angular space (Figure S4C).^89,90,91^ A flow chart of image processing is illustrated in Figure S11. Local filtering of the final map was carried out in CryoSPARC, map sharpening was carried out using DeepEMhancer.^100^ Resolution was determined from Fourier Shell Correction (FSC) using criterion of FSC = 0.143^114^. Map to model FSC curves were determined with the program EMDA^98^ (Figure S4D), with a resolution criterion of FSC = 0.5 In addition, directional FSC (dFSC)^115^ was used to evaluate the directional uniformity of the reconstructions, as reported in Table 1; in Figure S4E. Figures were generated from the map that is displayed with a spatially adaptive filter generated with a local resolution map (CryoSPARC).^89^

##### RNP image processing

Dose-weighted motion corrected sums were used for the estimation of the CTF utilizing CTFFIND4 wrapped within the RELION graphical user interface.^92^ Particles were picked in RELION using a reference-free method with a Laplacian-of-Gaussian (LoG) filter.^91^ Particles were extracted with a box size of 200 pixels for 2D classification. After multiple rounds of 2D classification, selected particles underwent 3D classification and refinement in RELION. Resolution was determined from Fourier Shell Correction (FSC) using criterion of FSC = 0.143^110^. Half-map FSC curves were determined in RELION postprocess (Figure S13).

#### Model building and refinement

The atomic models of human CRM1/Ran-GTP (PDB: 5DIS) and Rev/RRE (PDB: 4PMI) were docked into the cryo-EM map as a rigid body using UCSF ChimeraX.^94^ Residues 389–400 of CRM1 and residues 61–91 of Rev were modeled using Rosetta Enumerative Build^95^ followed by real space refinement.^58^ Except for CRM1 residues 389–400, CRM1 and Ran are undistinguishable from the previously solved crystal structure.^45^ We utilized the Rosetta program DRRAFTER^96^ to model the 39 nucleotides (119–154, 171–174) of the RRE that are ordered in the NEC, inputting the stem IIB portion of the RRE from the Rev/RRE dimer co-crystal structure^20^ together with RNA SHAPE data.^29^ The first 11 and the last 30 amino acids of Rev were not observed. The _MBP_Nup214_1916–2033_ construct is observable at higher contour levels and agrees with rigid body docking of the crystal structure of CRM1-SPN1-RanGTP-_MBP_Nup214 that the Nup214 peptide is bound to each CRM1 subunit (Figure S12).^45^ The modeled complex went through multiple rounds of refinement in real space using Phenix and manual adjusting using COOT.^58,97^ Map-to-model FSC plot was generated using EMDA.^98^ Visualizations of the atomic model were generated in UCSF ChimeraX.^94^

#### Dual luciferase assays

Rev reporter activities were measured in luciferase assays utilizing HEK293T cells transfected with Rev expression and RRE_234_ reporter constructs. HEK293T cells were co-transfected in 96-well plates with 0.5 ng of Rev wild-type or mutant plasmid, 49.5 mg of RRE_234_ reporter plasmid (pCMV gagpol-IRES FFL-RRE) and 0.5 ng of the reference plasmid pNL1.1.TK [Nluc/TK] (Promega, N1501) using PolyJet (SignaGen) following the manufacturer’s instructions. All assays were performed in biological triplicates. After 24 hours of transfection, the cells were lysed in 1x Passive Lysis Buffer (Promega), and firefly luciferase and NanoLuc luciferase activities were measured with the Promega Nano-Glo^®^ Dual-Luciferase^®^ Reporter Assay System and microplate reader (Ultra Evolution, Tecan). Rev-dependent firefly luciferase values were normalized to NanoLuc values. Rev expression levels were verified by Western blot using FLAG antibody (Sigma, F1804) with α-tubulin (Sigma, T5168) used as a loading control. IRES-FFL gene was inserted into pCMV gagpol-RRE_234_ vector as previously described by Gibson Assembly (New England Biosciences, US) for testing in Rev reporter assays.^63^

Rev reported activities were CRM1 mutants were measured in luciferase assyas utilizing BHK21 cells transfected with CRM1-expressing plasmids and Rev expression and RRE_234_ reporter constructs. BHK21 were co-transfected in 96-well plates with 20 ng CRM1 wild-type or mutant, 4 ng of Rev, 99 ng of RRE reporter plasmid (pCMV gagpol-IRES FFL-RRE) and 1 ng of the reference plasmid pNL1.1.TK [Nluc/TK] (Promega, N1501) using Lipofectamine^™^ 3000 (Invitrogen^™^) following the manufacturer’s instructions. All assays were performed in biological quadruplicates. After 24 hours of transfection, the cells were lysed in 1x Passive Lysis Buffer (Promega), and firefly luciferease and NanoLuc luciferase activities were measured with the Promega Nano-Glo^®^ Dual-Luciferase^®^ Reporter Assay System and microplate reader. Rev-dependent firefly luciferease values were normalized to NanoLuc values, and protein expression levels were monitored by Western blot.

#### Co-immunoprecipitation assays

Expi293 cells (Thermo Fisher Scientific) were transfected using the Expi293 transient expression system according to the manufacturer’s instructions. Cells were transfected with either 2xStrep-CRM1 (wild-type or variants), FLAG-SPN1, and HA-Ran Q69L 180, or with 2xStrep-CRM1 (wild-type or variants), FLAG-survivin and HA-Ran Q69L 180. Full-length human survivin with an N-terminal FLAG tag was cloned into the pcDNA4/TO vector between the NotI and XhoI restriction sites using a synthetic gene fragment (Table S2; Twist Bioscience, US). CRM1 was transfected at 500 ng/mL, Ran Q69L 180 at 250 ng/mL, and either SPN1 or surviving at 250 ng/mL, for a total of 1 μg/mL DNA per mL of suspension culture. Expi293 cells were lysed in lysis buffer (50 mM Tris pH 7.5, 150 mM NaCl_2_, 2 mM MgCl_2_, 0.5% Triton X-100 (v/v) 0.2% Na-deoxycholate (w/v)) supplemented with Halt^™^ protease Inhibitor for 30 minutes at 4°C by gentle rocking. Cleared cell lysates were incubated overnight at 4°C with Strep-Tactin XT 4Flow resin. The Strep-Tactin XT 4Flow resin was washed with lysis buffer without protease Inhibitor. Proteins were eluted with Buffer P (50 mM Tris pH 7.5, 150 mM NaCl_2_, 2 mM MgCl_2_, 0.5% Triton X-100 (v/v), 1x BXT buffer and eluates were analyzed by Western blotting with antibodies against the FLAG-tag, 2x Strep-tag, or CRM1.^99^

## Supplementary Material

MMC1

MMC2

MMC3

SUPPLEMENTAL INFORMATION

Supplemental information can be found online at https://doi.org/10.1016/j.molcel.2025.07.015.

## Figures and Tables

**Table 1.** Cryo-EM collection, refinement, and validation statistics for NEC

	ssDNA oligos	Polyamine
		
Data collection and processing		

Microscope	Titan Krios	Titan Krios
Magnification	EFTEM 105,000	EFTEM 105,000
Voltage (kV)	300	300
Electron exposure (e^−^/Å2)	66	68
Defocus range (μm)	−2.8	−2.8
Pixel Size (Å)	0.834	0.835
Micrographs collected (no.)	24,447	22,762
Symmetry imposed	C1	C1
Initial particle images (no.)	2,732,750	8,443,875
Final Particle images (no.)	260,885	
Map resolution (Å)	3.8	
FSC threshold	0.143	
Map resolution range (Å)	3.8–142.85	
**Refinement**	Data were combined	
Initial model used (PDB code)	PDB:5DIS (CRM1/Ran-GTP)/PDB: 4PMI (Rev dimer with stem IIB mimic)	
Model resolution (Å)	7.9	
FSC threshold range (Å)	0.5	
Model resolution range	3.8–142.85	
Map sharpening B factor (Å)	deepEMhancer	
**Model composition**		
Non-hydrogen atoms	21,963	
Protein residues	2,603	
Nucleotide	39	
**B-factors (Å^2^)**		
Protein	325	
**R.m.s deviations**		
Bond lengths (Å)	0.003	
Bond angles (°)	0.76	
**Validation**		
MolProbity score	1.94	
Clashscore	18.82	
Poor Rotamers (%)	0	
**Ramachandran plot**		
Favored (%)	97.03	
Allowed (%)	2.89	
Disallowed (%)	0.08	

**Table 2.** Cryo-EM collection statistics for the RNP

	Rev/RRE355
	
Data collection and processing	

Microscope	Glacios Arctica
Magnification	36,000
Voltage (kV)	200
Electron exposure (e−/Å^2^)	95
Defocus range (μm)	−2.8
Pixel Size (Å)	1.1505
Micrographs collected (no.)	3,976
Symmetry imposed	C1
Initial particle images (no.)	860,279
Final Particle images (no.)	126,797
Map resolution (Å)	10.02
FSC threshold	0.143
Map resolution range (Å)	10.02–230.62

**KEY RESOURCES TABLE:** 

REAGENT or RESOURCE	SOURCE	IDENTIFIER

Antibodies		

Mouse monoclonal ANTI-FLAG	Sigma-Aldrich	F1804; RRID: AB_262044
Mouse monoclonal ANTI-GAPDH	Proteintech	600004-1-lg; RRID: AB_2107436
Mouse monoclonal ANTI-α-Tubulin	Sigma-Aldrich	T5168; RRID: AB_477579
Rabbit Monoclonal ANTI-CRM1	Thermo Fisher Scientific	703569; RRID:AB_2784588
Mouse Monoclonal HRP-conjugated ANTI-Flag	Invitrogen	MA1-91878-HRP; RRID: AB_2537626

Bacterial and virus strains		

Stellar^™^ Competent Cells	Takara	636763
BL21(DE3) Competent	New England Biolabs	C2527H
BL21(DE3)pLysS	Invitrogen	C606010

Chemicals, peptides, and recombinant proteins		

Q5^®^ High-Fidelity 2X Master Mix	New England Biosciences	M0492S
In-Fusion^®^ Snap Assembly Master Mix	Takara Bio Inc.	638947
Phusion^™^ High-Fidelity DNA Polymerase	New England Biosciences	M0530S
Gibson Assembly^®^ Master Mix	New England Biosciences	E2611
Fisher BioReagents^™^ 2XYT Broth	Fisher Scientific	BP97432
SYBR^™^ Gold Nucleic Acid Gel Stain	Invitrogen^™^	S11494
FreeStyle^™^ 293 Expression System	Invitrogen^™^	K900001
FreeStyle^™^ 293 Expression Medium	Invitrogen^™^	12338018
Expi293^™^ Expression System Kit	Thermo Fisher Scientific	A14635
DMEM/High Glucose	Cytvia	SH30022.01
Fetal Bovine Serum (Adherent HEK Cells)	Gibco	A52568-01
Penicillin Streptomycin	Gibco	15140–122
Lipofectamin 3000 Transfection Reagent	Invitrogen	L3000008
PolyJet^™^ In Vitro DNA Transfection Reagent	SignaGen Laboratories	SL100688
Promoter-Driven Control NanoLuc^®^ Luciferase Vector (pNL1.1.TK)	Promega	N1501
Passive Lysis 5x Buffer	Promega	E1941
Nano-Glo^®^ Dual-Luciferase^®^ Reporter Assay System	Promega	N1610
Halt^™^ Protease Inhibitor Cocktail (100X)	Thermo Scientific^™^	78429
RosettaTM(DE3) pLysS	Novagen	70956–3
Amylose Resin	New England Biosciences	E8021L
Pierce^™^ Bovine Serum Albumin Standard Pre-Diluted Set	Thermo Scientific^™^	23208
Strep-Tactin^®^XT 4Flow^®^ resin	IBA Lifesciences, GmbH	2-5010-025
Buffer BXT (10x)	IBA Lifesciences, GmbH	2-1042-025
HisPur^™^ Ni-NTA Resin	Thermo Scientific^™^	88222
HIV-1 Rev HXB3 wild type	Daugherty et al.^22^	N/A
HIV-1 Rev HXB3 L60R	Daugherty et al.^22^	N/A
RanQ69L	Booth et al.^54^	N/A

Deposited data		

HIV-1 NEC Map	This paper	EMDB-42494
HIV-1 NEC Model	This paper	PDB: 8URJ
HIV-1 Rev/RRE RNP Map	This paper	EMDB-70347
CRM1-RanGTP/SPN1/Nup214	Port et al.^45^	PDB: 5DIS
HIV-1 Rev/Stem IIB mimic	Jayaraman et al.^20^	PDB: 4PMI

Experimental models: Cell lines		

HEK293T cells	ACTT Cat# CRL-3216	N/A
BHK-21 cells	ACTT Cat# CCL-10	N/A

Oligonucleotides		

Primers See Table S4	IDT	N/A

Recombinant DNA		

Gene Fragments See Table S3	IDT/Twist Biosciences	N/A

Software and algorithms		

SerialEM	Mastronarde^87^	https://bio3d.colorado.edu/SerialEM/; RRID: SCR_019198
MotionCor2	Zheng et al.^88^	https://emcore.ucsf.edu/cryoem-software; RRID: SCR_016499
cryoSPARC	Punjani et al.^89^	https://cryosparc.com; RRID: SCR_016501
RELION 2.0	Fernandez-Leiro and Scheres^90^	https://cam.ac.uk/relion; RRID: SCR_016274
RELION 3.0	Zivanov et al.^91^	https://cam.ac.uk/relion; RRID: SCR_016274
CTFFIND4	Rohou and Grigorieff^92^	https://elia.org/ctffind4; RRID: SCR_016732
UCSF pyem	Asarnow et al.^93^	https://github.com/asarnow/pyem; https://doi.org/10.5281/zenodo.3576630
ChimeraX	Goddard et al.^94^	https://www.cgl.ucsf.edu/chimerax/download.html; RRID: SCR_015872
Rosetta Enumerative Build	Das^95^	https://www.rosettacommons.org/home; RRID: SCR_015701
DRRAFTER	Kappel^96^	https://www.rosettacommons.org/home; RRID: SCR_015701
COOT	Casañal^97^	http://www2.mrc-lmb.cam.ac.uk/personal/pemsley/coot/; RRID: SCR_014222
PHENIX	Liebschner, et al.^58^	https://www.phenix-online.org/; RRID: SCR_014224
PHENIX	Adams et al.^59^	https://www.phenix-online.org/; RRID: SCR_014224
EMDA	Warshamanage et al.^98^	https://pypi.org/project/emda/
cis-TEM	Grant et al.^99^	https://cistem.org/; RRID: SCR_016502
deepEM-hancer	Sanchez-Garcia et al^100^	https://github.com/rsanchezgarc/deepEMhancer
Prisim 8	GraphPad	http://www.graphpad.com/; RRID: SCR_002798

## Highlights

- Rev binds an uncharacterized CRM1 site
- Rev dimer drives CRM1 dimer
- RRE engages CRM1/Ran-GTP
