# Supplementary material for: The HIV-1 nuclear export complex reveals the role of RNA in CRM1 cargo recognition
Source: Mol Cell. Author manuscript; Available in PMC 2025 Sep 4. (PMC12410093; doi:10.1016/j.molcel.2025.07.015)
Supplement: MMC1 [file NIHMS2104704-supplement-MMC1.pdf]

Molecular Cell, Volume *85*

Supplemental information

The HIV-1 nuclear export complex reveals the role of RNA in CRM1 cargo recognition

Amber M. Smith, Yang Li, Arianna Velarde, Yifan Cheng, and Alan D. Frankel

Supplemental Information

The HIV-1 Nuclear Export Complex Reveals the Role of RNA in CRM1 Cargo Recognition

Authors: Amber M. Smith[1], Yang Li[1], Arianna Velarde[2], Yifan Cheng[1,3,*], Alan D. Frankel[1,*]

Figure S1

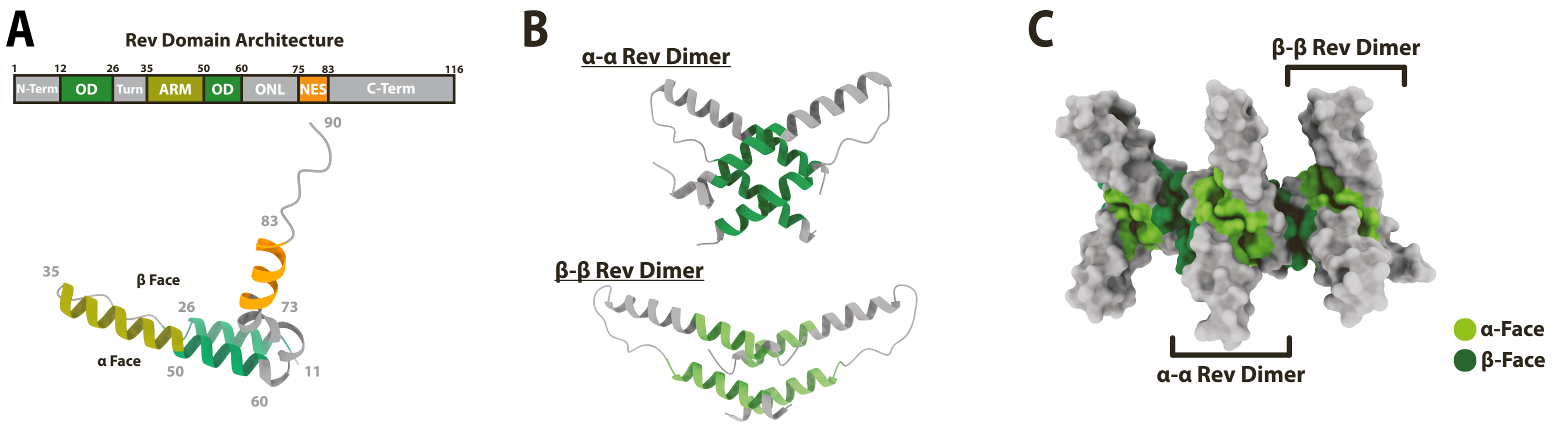
A
Rev Domain Architecture
1
12
26
35
50
60
75
83
116
N-Term
OD
Turn
ARM
OD
ONL
NES
C-Term
90
83
35
β Face
26
73
α Face
50
11
60
B
α-α Rev Dimer
β-β Rev Dimer
C
β-β Rev Dimer
α-α Rev Dimer
α-Face
β-Face

Figure S1: Structural organization of HIV-1 Rev (related to Figures 1 and 2).

(A) Domain architecture of HIV-1 Rev (strain HXB3). Rev consists of three primary functional domains: a bipartite oligomerization domain (OD; green) flanking the arginine rich motif (ARM; yellow) and a C-terminal nuclear export sequence (NES). Additional flanking regions include N- and C-terminal domains, the turn region, and the OD-NES-Linker (ONL). Rev adopts a hairpin conformation, with the NES extending beyond the hairpin. Both the α- and β-faces of the Rev hairpin are critical for Rev function.

(B) Rev can self-associate via both faces of its hairpin structure forming a V-like topology. Crystal structures have revealed that Rev dimers can form via interactions on either the α-face (dark green, PDB: 3LPH[S1]) or β-face (light green; PDB 2X7L[S2]), however, apart from the Rev filament, no structure has yet been observed in which both faces participate in dimerization simultaneously.

(C) Given that Rev can dimerize through either face and that biochemical data indicate multiple Rev molecules bind the RRE, it has been long hypothesized that Rev forms higher-order oligomeric assemblies, supported by interactions observed in crystal contacts and in the Rev filament.

Figure S2

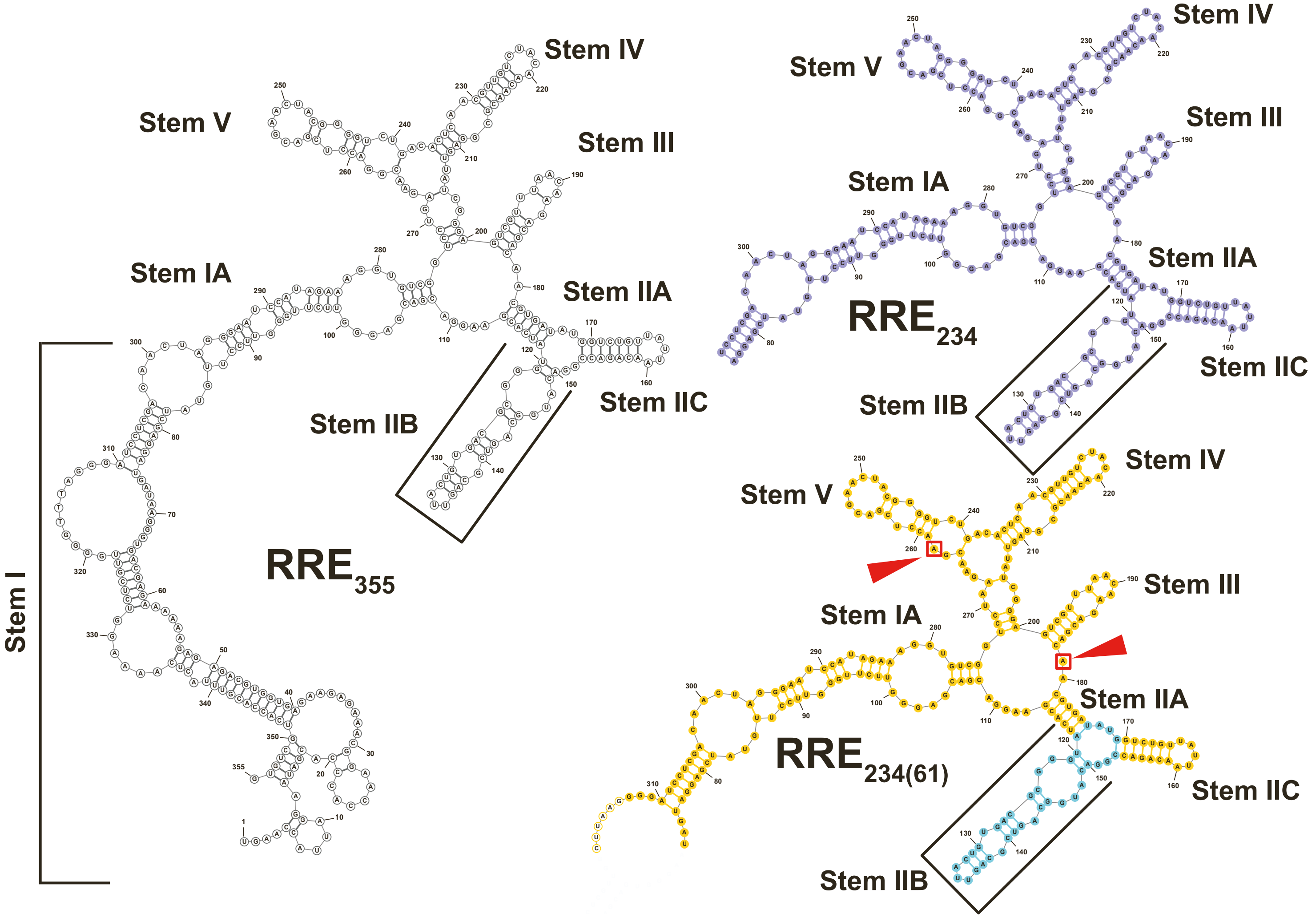

Stem IV
Stem V
Stem III
Stem IA
Stem IIA
Stem IIB
Stem IIC
Stem I
RRE355
RRE234
RRE234(61)

Figure S2: Secondary structure of the HIV-1 SF-2 RRE variants (related to Figure 1 and STAR Methods).

Sequence of the full-length HIV-1 RRE_{355}, isolate SF-2, is shown in gray. Sequence of RRE_{234} (the minimal RRE that supports Rev function and virus replication) is shown in purple. Sequence of $RRE_{234(61)}$ in the truncated context is shown with stem IIB in cyan. Compensatory mutations in RRE_{61} responsible for viral resistance to a Rev NES dominant negative mutant (G262 and A181) are highlighted with red arrows[S3] . Position 181 is a guanosine in the NL4-3 isolate but was already an adenosine in the SF-2 isolate. An adenosine at position 269 in the NL4-3 RRE is critical for RRE_{61} resistance, therefore G269 was mutated to an adenosine in the SF-2 RRE.

Figure S3

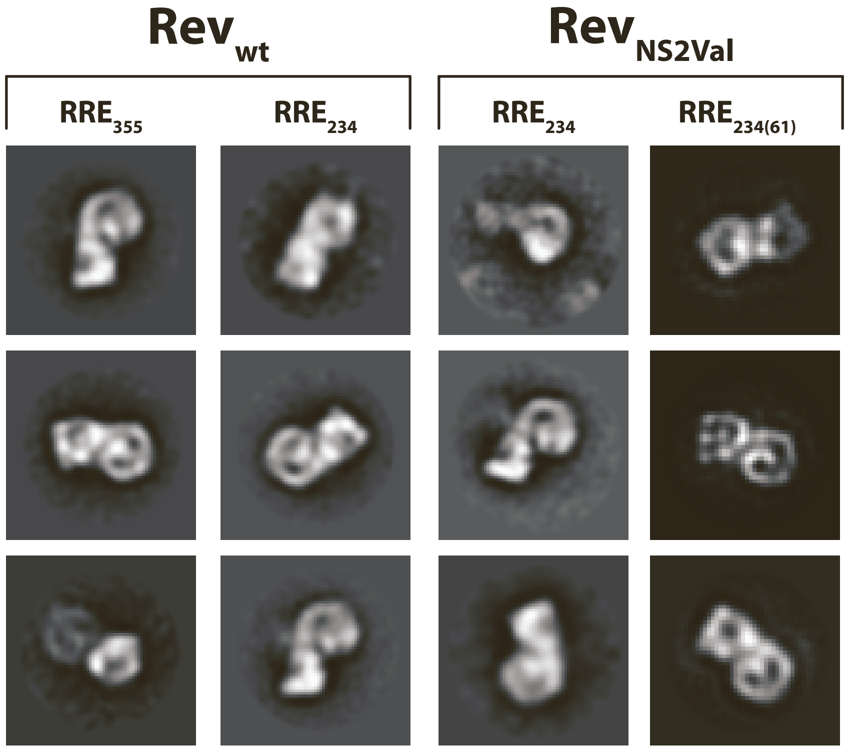

Figure S3: Representative negative stain 2D class averages of NECs assembled with the different Rev and RRE variants used in this study (related to Figure 1 and STAR Methods).
Complexes were assembled with either Rev_{wt} or Rev_{NS2Val} and either RRE_{355}, RRE_{234} or $RRE_{234(61)}$.

Figure S4

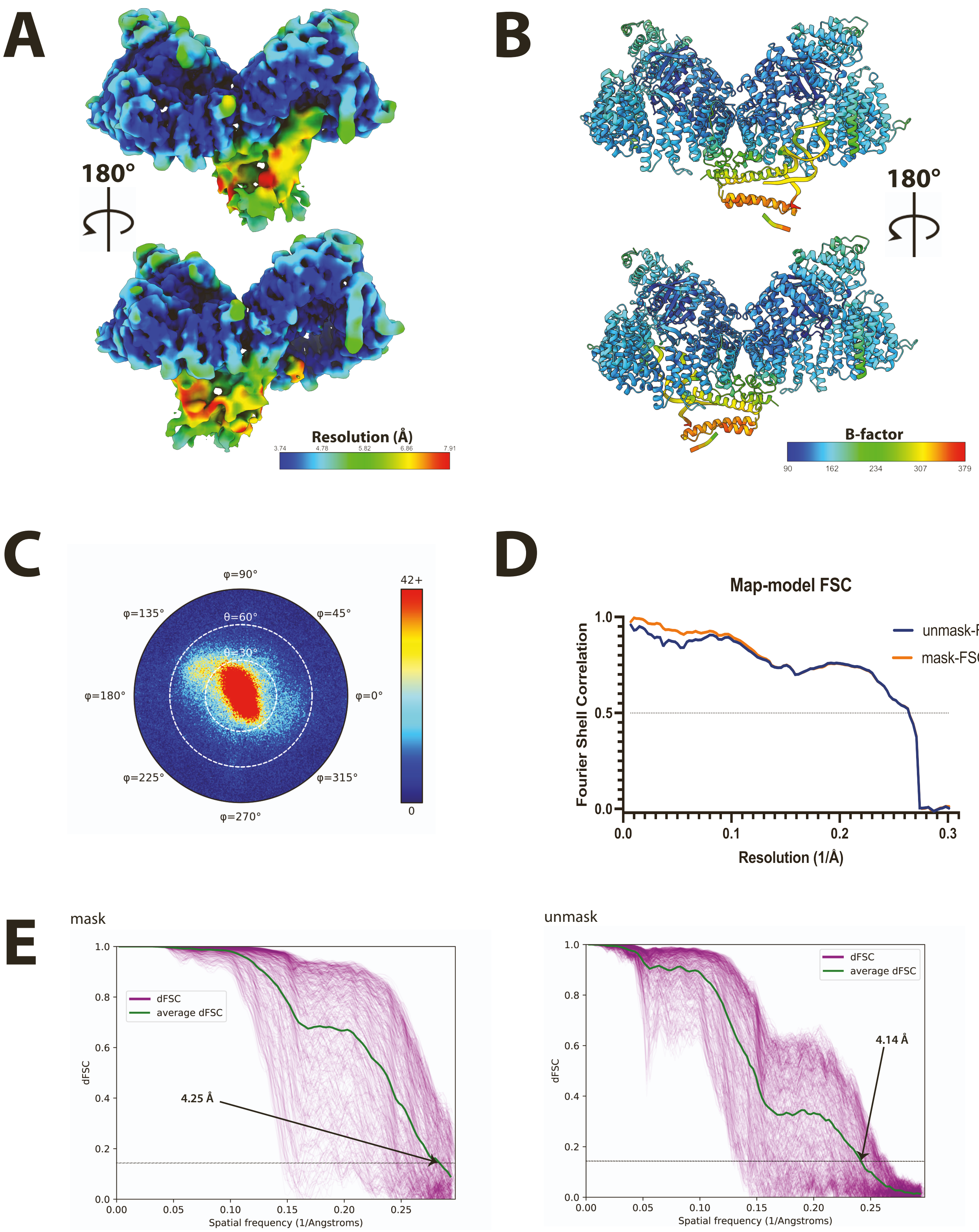
A
180°
Resolution (Å)
3.74
4.78
5.82
6.86
7.91
B
180°
B-factor
90
162
234
307
379
C
φ=90°
φ=45°
φ=135°
θ=60°
θ=30°
φ=180°
φ=0°
φ=225°
φ=315°
φ=270°
42+
0
D
Map-model FSC
unmask-FSC
mask-FSC
Fourier Shell Correlation
Resolution (1/Å)
E
mask
dFSC
average dFSC
4.25 Å
Spatial frequency (1/Angstroms)
unmask
4.14 Å

Figure S4: Cryo-EM map quality and metrics for NEC (related to Figure 1).
(A) Local resolution estimate of the NEC reconstruction (cryoSPARC[S4] , local filtered map).
(B) Refined NEC model colored according to B-factor.
(C) Euler angle distribution of all particles used in the final 3D reconstruction (cisTEM[S5]).
(D) Map to model FSC calculated by EMDA[S6] .
(E) Directional FSC curves were estimated as previously described[S7] . Left is with a mask, right is without.

Figure S5

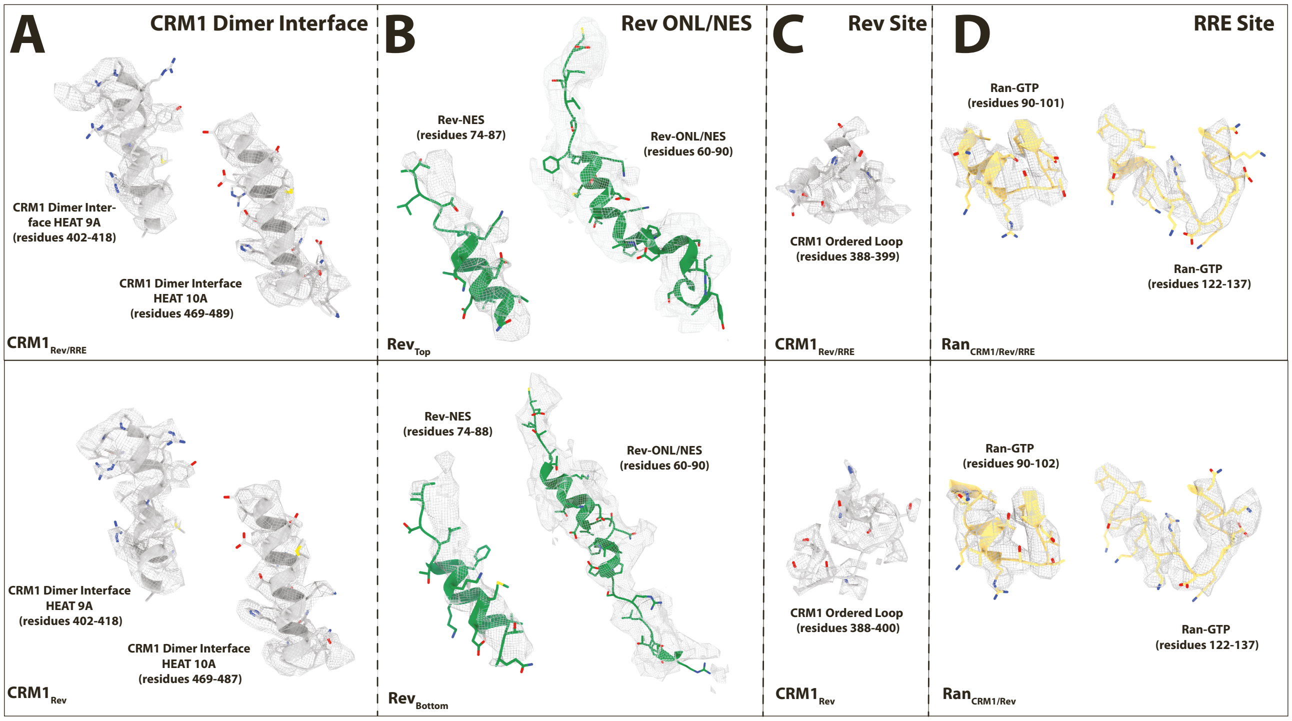
A
CRM1 Dimer Interface
CRM1 Dimer Inter-
face HEAT 9A
(residues 402-418)
CRM1 Dimer Interface
HEAT 10A
(residues 469-489)
CRM1 Rev/RRE
B
Rev ONL/NES
Rev-NES
(residues 74-87)
Rev-ONL/NES
(residues 60-90)
Rev Top
C
Rev Site
CRM1 Ordered Loop
(residues 388-399)
CRM1 Rev/RRE
D
RRE Site
Ran-GTP
(residues 90-101)
Ran-GTP
(residues 122-137)
Ran CRM1/Rev/RRE
CRM1 Dimer Interface
HEAT 9A
(residues 402-418)
CRM1 Dimer Interface
HEAT 10A
(residues 469-487)
CRM1 Rev
Rev-NES
(residues 74-88)
Rev-ONL/NES
(residues 60-90)
Rev Bottom
CRM1 Ordered Loop
(residues 388-400)
CRM1 Rev
Ran-GTP
(residues 90-102)
Ran-GTP
(residues 122-137)
Ran CRM1/Rev

Figure S5: Representative cryo-EM densities from selected structural features (related to Figures 1, 3, 5 and 6).

Representative segments of the cryo-EM map highlight structural features described in the text.
(A) The two helices that make up the CRM1 dimer interface and contain the species-specific residues (sharpened map from DeepEMHancer[S8]).
(B) Rev NES and NES/ONL that interacts with CRM1 Map of NES alone is the sharpened map from DeepEMHancer[S8]. Map of ONL/NES is the locally filtered map from CryoSPARC[S4] .
(C) The ordered loop of CRM1 (residues 388-400), which becomes ordered when bound to the Rev/RRE RNP. Map is the locally filtered map from CryoSPARC[S4] .
(D) Loops from Ran-GTP that contain the residues (R95, K130, K132 and K134) that interact with stem IIB (sharpened map from DeepEMHancer[S8]).

Figure S6

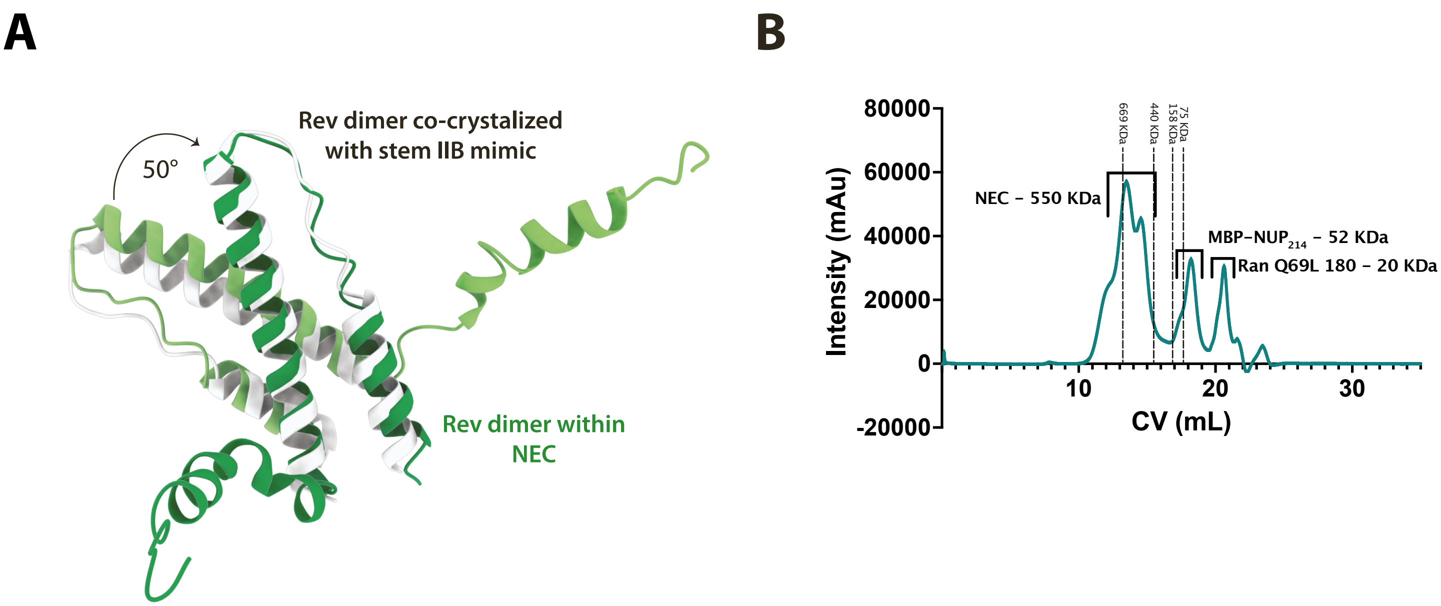

A
50°
Rev dimer co-crystalized with stem IIB mimic
Rev dimer within NEC
B
80000
60000
40000
20000
0
-20000
Intensity (mAu)
10
20
30
CV (mL)
669 KDa
440 KDa
158 KDa
75 KDa
NEC – 550 KDa
MBP–NUP$_{214}$ – 52 KDa
Ran Q69L 180 – 20 KDa

Figure S6: The Rev dimer interface and multivalent Rev binding within the NEC were previously described (related to Figure 2).

(A) Overlap of the NEC Rev dimer (dark and light green) with the Rev dimer co-crystallized with a stem IIB mimic (gray; PDB: 4PMI). Both Rev dimers exhibit the same dimer crossing angle, highlighting that stem IIB drives the dimer crossing angle of the Rev dimer at this position.

(B) The NEC used in the current study was analyzed using a Superose 6 Increase column calibrated with molecular weight standards to determine its stoichiometry. The elution profile revealed two peaks, both corresponding to a molecular weight between 440 kDa and 669 kDa, with an estimated NEC size of ~550 kDa. The complex was assembled in the presence of excess MBP-Nup214 and RanQ69L180-GTP to promote complex formation and stability.

Figure S7

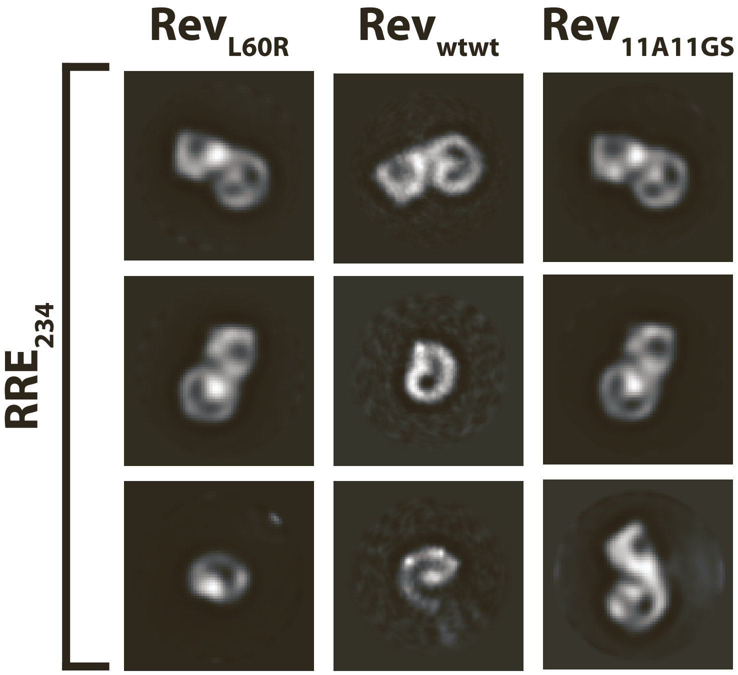

Figure S7: Representative negative stain 2D class averages of Rev variants to assess NEC assembly efficiency (related to Figure 5).

NECs were assembled using HIV-1 RRE_{234} and one of three Rev variants: the monomeric oligomerization-deficient mutant (L60R), the linked wild-type Rev dimer (Rev_{wtwt}), or the linked Rev dimer with a helical ONL in the N-terminal position and a flexible ONL in the C-terminal position ($Rev_{11A11GS}$). Representative 2D class averages demonstrate differences in complex assembly efficiency across the Rev variants as judged by completeness of CRM1 dimer formation.

Figure S8

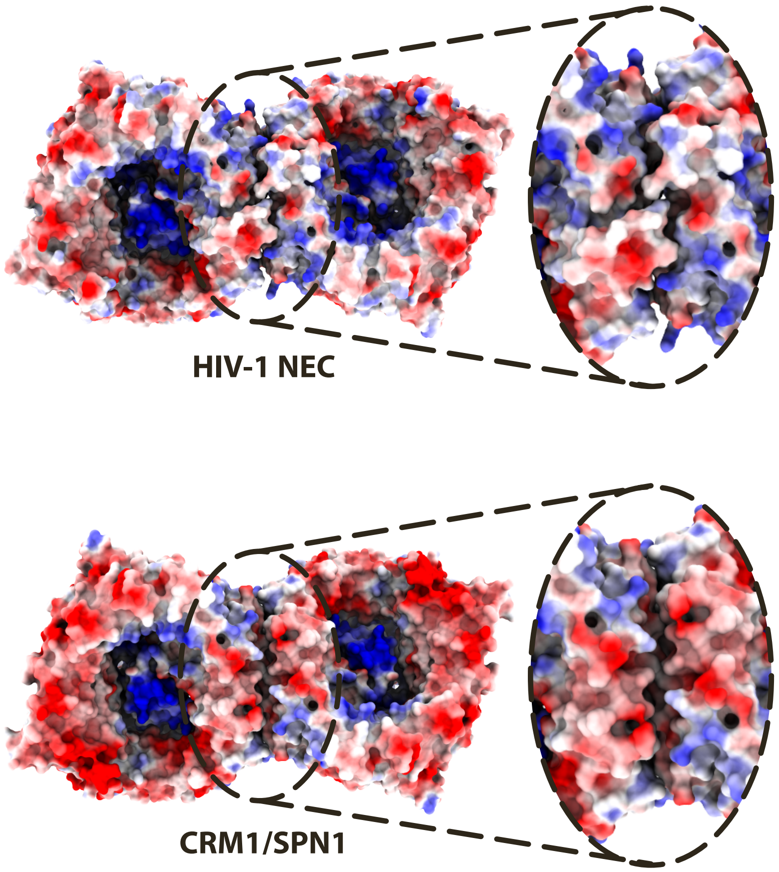

Figure S8: Conformations of the loop between HEAT repeats 8 and 9 modulate the electrostatics of the CRM1 dimer interface (related to Figure 3).
Electrostatic surface representation of the CRM1 dimer within the NEC (top) shows the loop between HEAT repeats 8 and 9 (residues 389-400) in an ordered conformation, forming a hydrophobic surface that accommodates Rev binding. In contrast, the CRM1 dimer modeled using the SPN1/CRM1 complex (PDB:5DIS; bottom), in which this loop is disordered, exhibts increased negative charge in this region (red is negative, blue is positive), suggesting that loop ordering remodels the electrostatic landscape to favor Rev engagement.

Figure S9

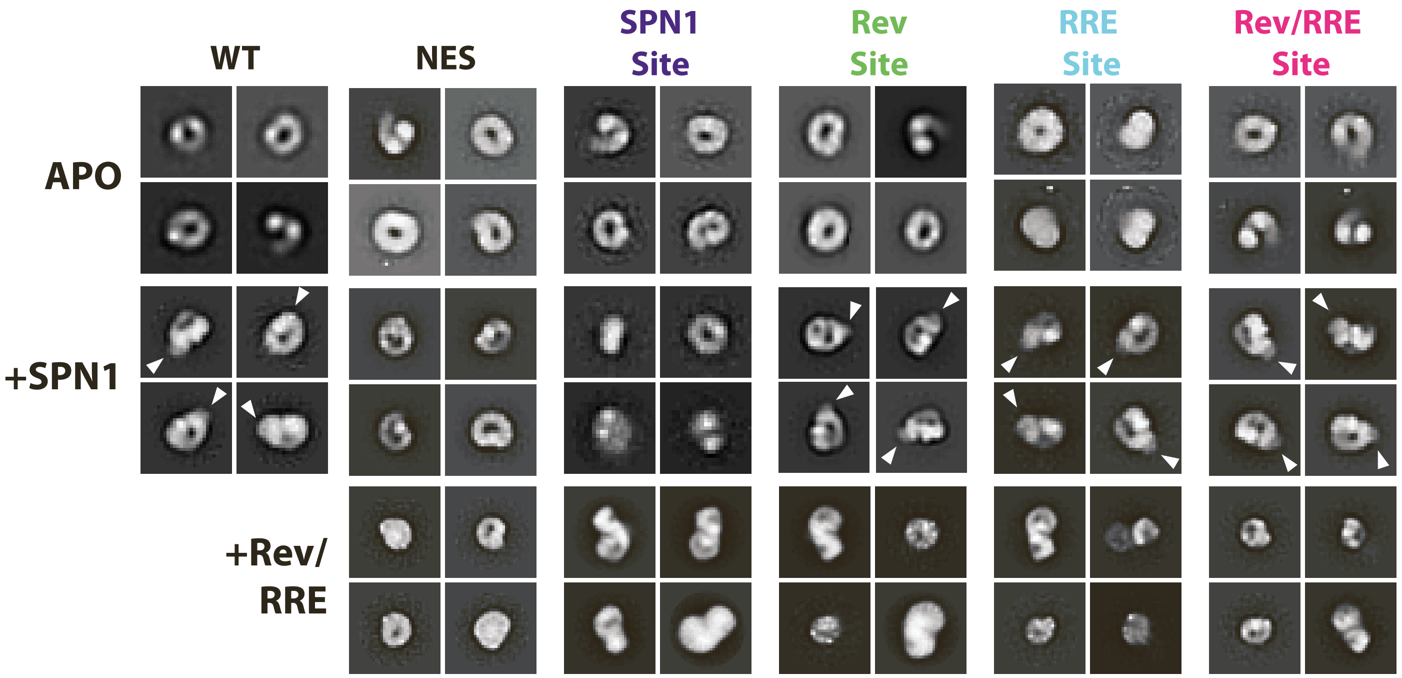
WT
NES
SPN1 Site
Rev Site
RRE Site
Rev/RRE Site
APO
+SPN1
+Rev/ RRE

Figure S9: Representative negative stain 2D class averages of CRM1 variants in the apo state or bound to either SPN1 or the Rev/RRE RNP to assess overall architecture and efficiency of complex assembly (related to Figure 4).

Shown are representative 2D class averages of apo CRM1 (wild-type and $SPN1_1$, Rev_1, RRE_1, and Rev/RRE_1), CRM1/Ran-GTP bound to SPN1 (using the same CRM1 constructs as in the apo state), and CRM1/Ran-GTP bound to the HIV-1 Rev/RRE_{234} RNP (using the same CRM1 constructs as in the apo state). This comparison allows assessment of structural differences and complex assembly efficiency across each CRM1 variant.

Figure S10

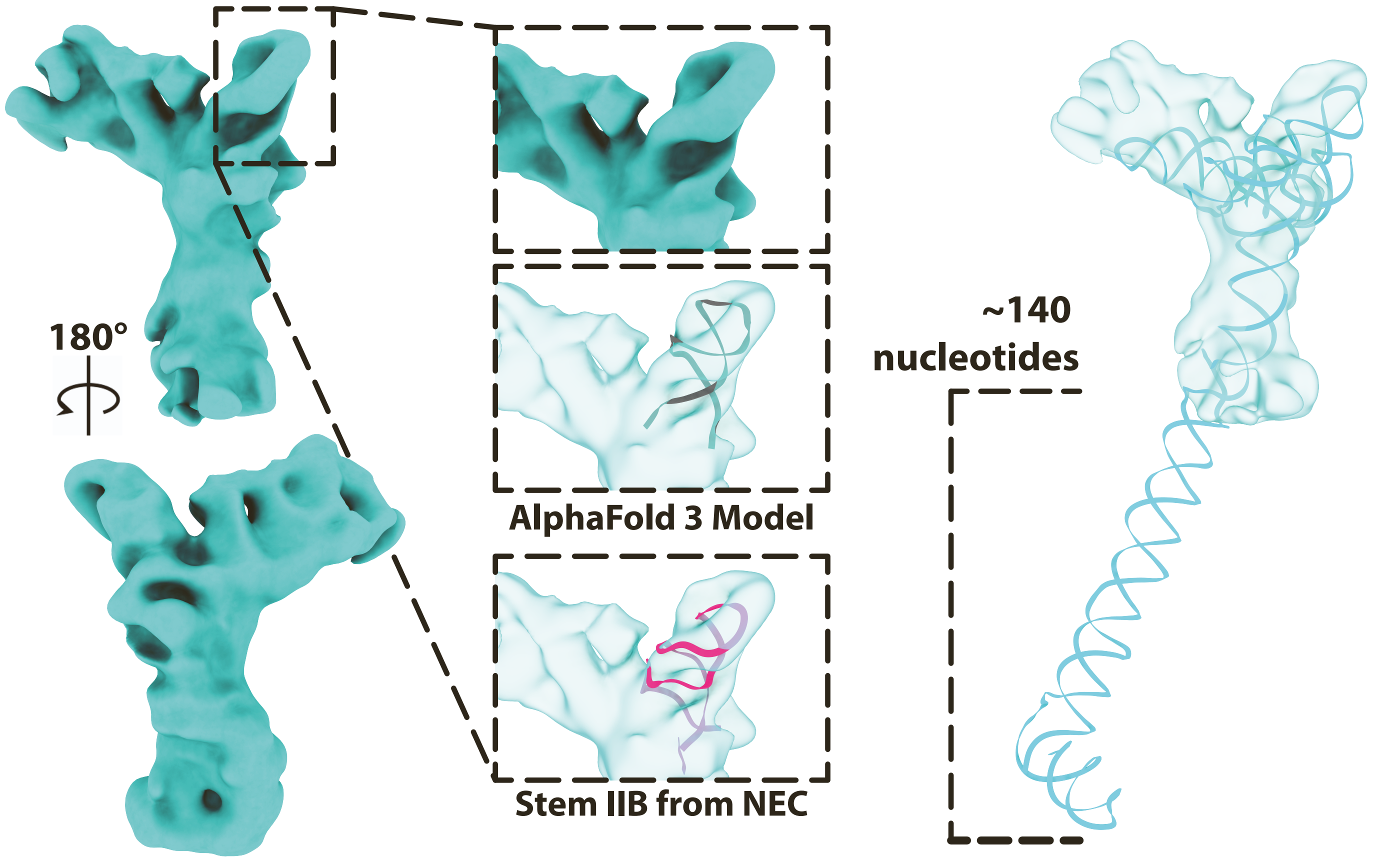

Figure S10: Cryo-EM reconstruction of the Rev/RRE$_{355}$ RNP (related to Figures 1 and 2). Cryo-electron microscopy (cryo-EM) reconstruction of the Rev/RRE$_{355}$ ribonucleoprotein (RNP) complex reveals an extended conformation for the RRE, supporting the flexibility observed in the NEC reconstruction. It was not possible to assign all loops to specific regions of the reconstruction, but one region exhibits clear secondary structure features consistent with the length and helical pitch of stem IIB, matching both an AlphaFold 3 model and the stem IIB within the NEC. The highest-scoring AlphaFold 3 model shares a similar overall architecture to our reconstruction; however, the latter lacks sufficient density to assign the highly elongated stem I. This figure shows a cryo-EM map resulting from 3D classification during data processing.

Figure S11

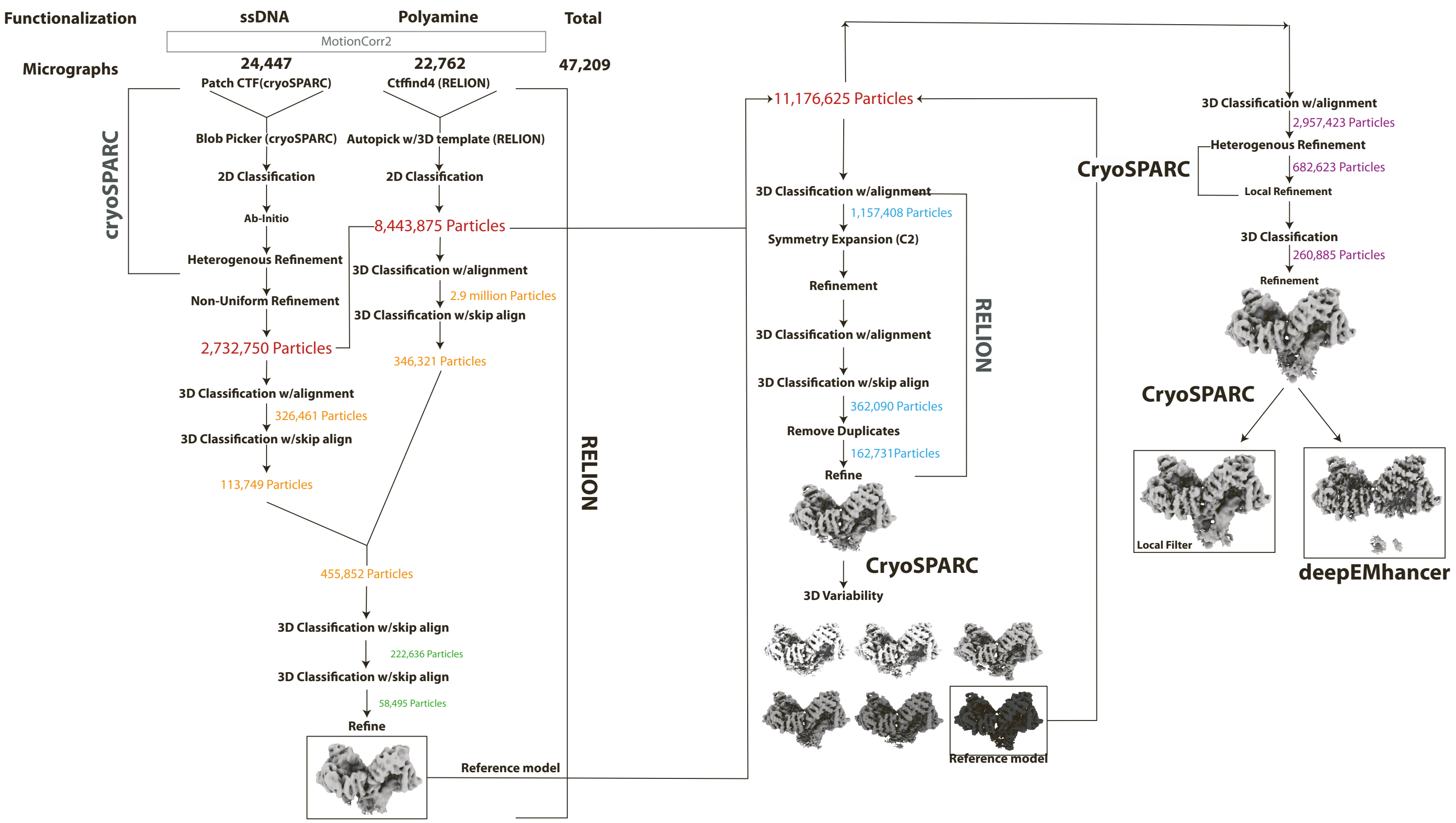
Functionalization
ssDNA
Polyamine
Total
MotionCorr2
Micrographs
24,447
22,762
47,209
Patch CTF(cryoSPARC)
Ctffind4 (RELION)
cryoSPARC
Blob Picker (cryoSPARC)
Autopick w/3D template (RELION)
2D Classification
2D Classification
Ab-Initio
8,443,875 Particles
Heterogenous Refinement
3D Classification w/alignment
Non-Uniform Refinement
2.9 million Particles
3D Classification w/skip align
2,732,750 Particles
346,321 Particles
3D Classification w/alignment
326,461 Particles
3D Classification w/skip align
113,749 Particles
RELION
455,852 Particles
3D Classification w/skip align
222,636 Particles
3D Classification w/skip align
58,495 Particles
Refine
Reference model
11,176,625 Particles
3D Classification w/alignment
1,157,408 Particles
Symmetry Expansion (C2)
Refinement
3D Classification w/alignment
RELION
3D Classification w/skip align
362,090 Particles
Remove Duplicates
162,731Particles
Refine
CryoSPARC
3D Variability
Reference model
CryoSPARC
3D Classification w/alignment
2,957,423 Particles
Heterogenous Refinement
682,623 Particles
Local Refinement
3D Classification
260,885 Particles
Refinement
CryoSPARC
Local Filter
deepEMhancer

Figure S11: Cryo-EM data processing workflow (related to Figure 1 and STAR Methods). Schematic flow-chart representing the image processing approach for the NEC. Thumbnail images of the NEC after refinement at different steps are shown and how each reconstruction was then used as initial reference model to re-process the initial particle stack to aid in correct particle alignments resulting in an increased overall resolution of the complex.

Figure S12

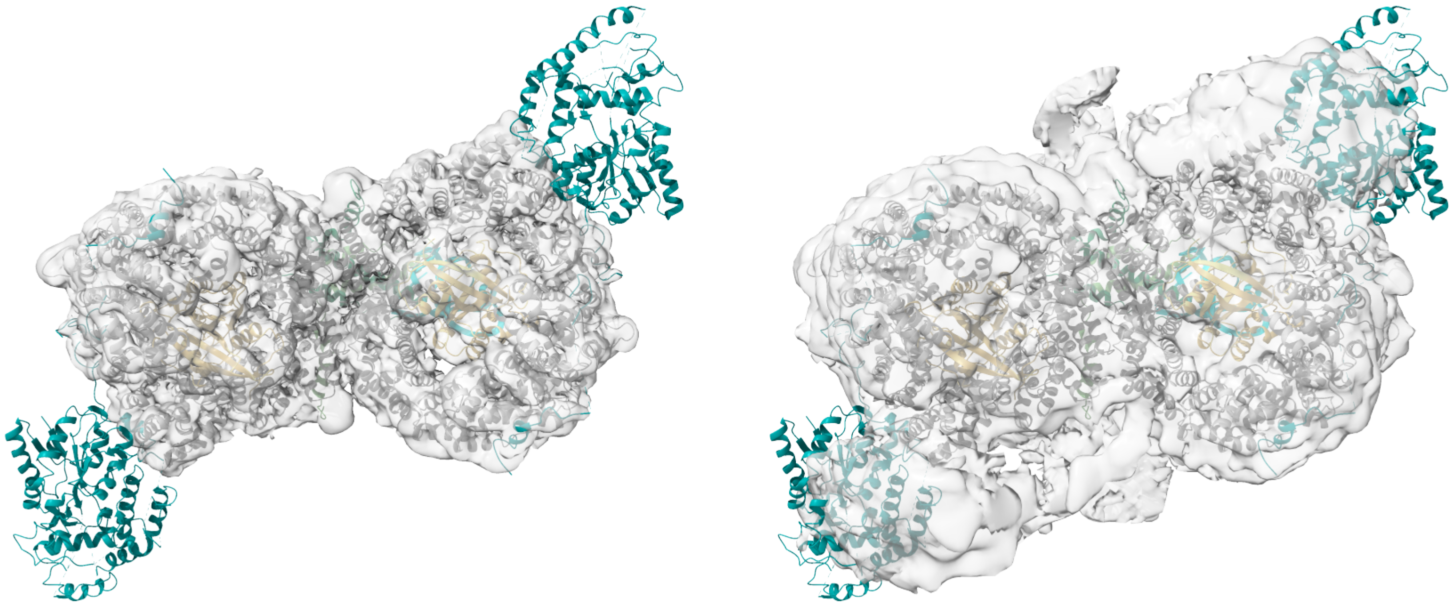

Figure S12: MBPNup214$_{1916\text{-}2033}$ binds to the NEC complex (related to Figure 1).
Increasing the contour level reveals density for the MBP portion of MBPNup214$_{1916\text{-}2033}$ and validates the presence of the Nup214 peptide bound to each CRM1 subunit. Left: The NEC shown at contour levels related to the resolution of the complex. Right: Same map as on the left with increased contour levels.

Figure S13

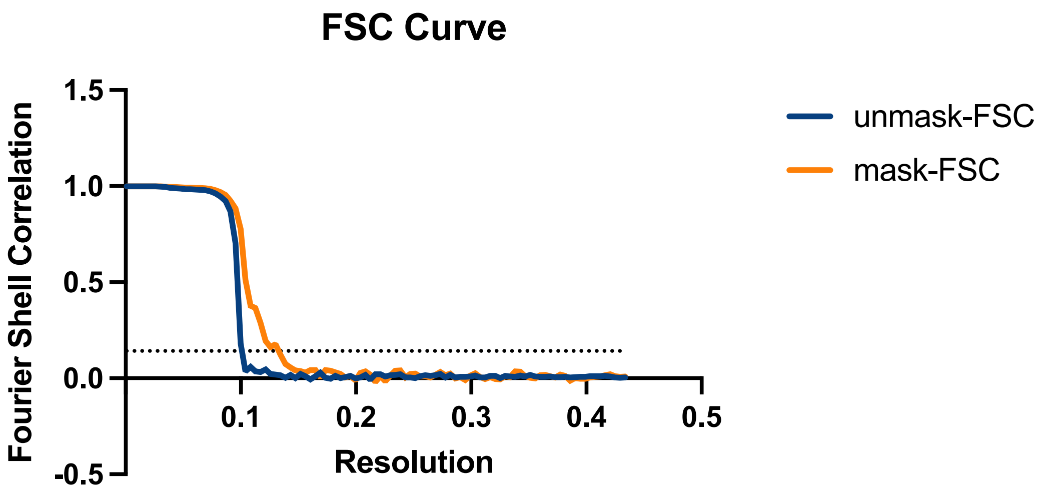

FSC Curve
Fourier Shell Correlation
1.5
1.0
0.5
0.0
-0.5
0.1
0.2
0.3
0.4
0.5
Resolution
unmask-FSC
mask-FSC

Figure S13: Half-map FSC curve for Rev/RRE_{355} reconstruction (related to STAR Methods)
Half-map FSC curve calculated by RELION[S9] **.**

Figure S14

Figure 4B:

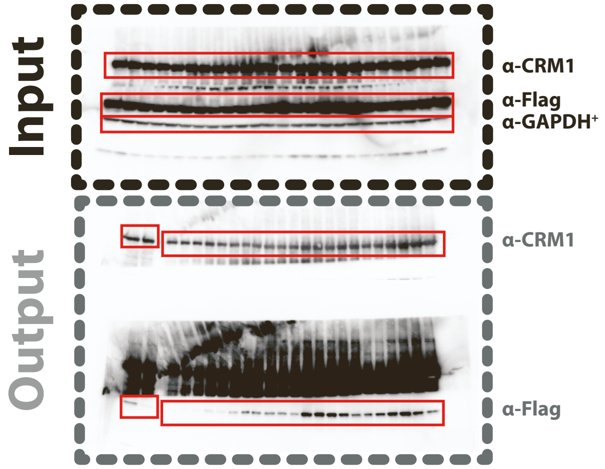

Figure 4C:

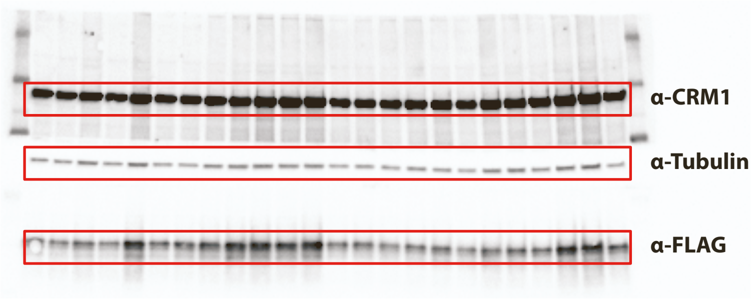

Figure 5B:

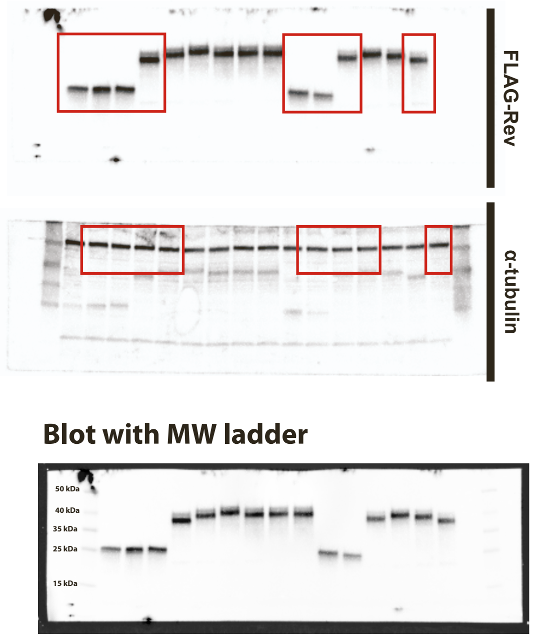

Figure 7:

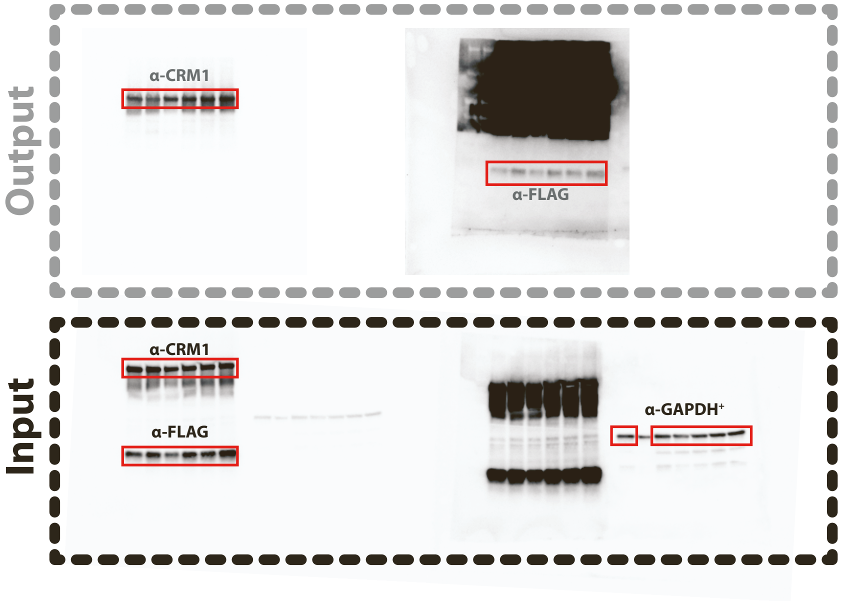

Figure S14: Uncropped Western blots corresponding to Figures 4B, 4C, 5B and 7.

Table S3: Sequences, functional groups, micrograph count and tilt angle for each data set collected. (related to STAR Methods)

Functionalization	Sequence/Functional Group	Micrograph total (no.)	Collection Tilt (°)
ssDNA #1	5AmMC6/GG TAC CCG GGG ATC G	2,224	0
ssDNA #2	5AmMC6/GGT ACN NNN GGA TCG	2,821	0
ssDNA #3	5AmMC6/GGT NNN NNN NNA TCG	2,776	0
ssDNA #4	5AmMC6/NNN NNN NNN N	3,206	0
ssDNA #5	5AmMC6/NNN NNN NNN NNN NNN	3,169	0
ssDNA #5	5AmMC6/NNN NNN NNN NNN NNN	7,286	25
ssDNA #6	5AmMC6/NNN NNN NNN NNN NNN NNN NN	2,965	0
Polyamine	C2H4(NH2)2	7,720	0
Polyamine	C2H4(NH2)3	11,190	15
Polyamine	C2H4(NH2)4	3,852	30
	**Total**	**47,209**	

S1. Daugherty, M.D., Liu, B., and Frankel, A.D. (2010). Structural basis for cooperative RNA binding and export complex assembly by HIV Rev. Nat Struct Mol Biol *17*, 1337–1342. 10.1038/nsmb.1902.

S2. DiMattia, M.A., Watts, N.R., Stahl, S.J., Rader, C., Wingfield, P.T., Stuart, D.I., Steven, A.C., and Grimes, J.M. (2010). Implications of the HIV-1 Rev dimer structure at 3.2 A resolution for multimeric binding to the Rev response element. Proc. Natl. Acad. Sci. U.S.A. *107*, 5810–5814. 10.1073/pnas.0914946107.

S3. Hamm, T.E., Rekosh, D., and Hammarskjöld, M.-L. (1999). Selection and Characterization of Human Immunodeficiency Virus Type 1 Mutants That Are Resistant to Inhibition by the Transdominant Negative RevM10 Protein. J Virol *73*, 5741–5747. 10.1128/jvi.73.7.5741-5747.1999.

S4. Punjani, A., Rubinstein, J.L., Fleet, D.J., and Brubaker, M.A. (2017). cryoSPARC: algorithms for rapid unsupervised cryo-EM structure determination. Nat. Methods. 10.1038/nmeth.4169.

S5. Grant, T., Rohou, A., and Grigorieff, N. (2018). cisTEM, user-friendly software for single-particle image processing. eLife *7*, e35383. 10.7554/elife.35383.

S6. Warshamanage, R., Yamashita, K., and Murshudov, G.N. (2022). EMDA: A Python package for Electron Microscopy Data Analysis. J. Struct. Biol. *214*, 107826. 10.1016/j.jsb.2021.107826.

S7. Dang, S., Feng, S., Tien, J., Peters, C.J., Bulkley, D., Lolicato, M., Zhao, J., Zuberbühler, K., Ye, W., Qi, L., et al. (2017). Cryo-EM structures of the TMEM16A calcium-activated chloride channel. Nature *552*, 426–429. 10.1038/nature25024.

S8. Sanchez-Garcia, R., Gomez-Blanco, J., Cuervo, A., Carazo, J.M., Sorzano, C.O.S., and Vargas, J. (2021). DeepEMhancer: a deep learning solution for cryo-EM volume post-processing. Commun. Biol. *4*, 874. 10.1038/s42003-021-02399-1.

S9. Zivanov, J., Nakane, T., Forsberg, B.O., Kimanius, D., Hagen, W.J., Lindahl, E., and Scheres, S.H. (2018). New tools for automated high-resolution cryo-EM structure determination in RELION-3. eLife *7*, e42166. 10.7554/elife.42166.
